# Geographical and spatial variations in bowel cancer screening participation, Australia, 2015–2020

**DOI:** 10.1371/journal.pone.0288992

**Published:** 2023-07-20

**Authors:** Paramita Dasgupta, Jessica K. Cameron, Belinda Goodwin, Susanna M. Cramb, Kerrie Mengersen, Joanne F. Aitken, Peter D. Baade

**Affiliations:** 1 Cancer Research Centre, Cancer Council Queensland, Brisbane, Queensland, Australia; 2 School of Mathematical Sciences, Queensland University of Technology, Brisbane, Queensland, Australia; 3 Centre for Heath Research, University of Southern Queensland, Springfield, Queensland, Australia; 4 Australian Centre for Health Services Innovation & Centre for Healthcare Transformation, Queensland University of Technology, Brisbane, Queensland, Australia; 5 School of Public Health and Social Work, Queensland University of Technology, Brisbane, Queensland, Australia; 6 Centre for Data Science, Queensland University of Technology, Brisbane, Queensland, Australia; 7 School of Public Health, University of Queensland, Brisbane, Queensland, Australia; 8 Menzies Health Institute Queensland, Griffith University, Gold Coast Campus, Southport, Queensland, Australia; The University of Sydney, AUSTRALIA

## Abstract

**Background:**

Participation in bowel cancer screening programs remains poor in many countries. Knowledge of geographical variation in participation rates may help design targeted interventions to improve uptake. This study describes small-area and broad geographical patterns in bowel screening participation in Australia between 2015–2020.

**Methods:**

Publicly available population-level participation data for Australia’s National Bowel Cancer Screening Program (NBCSP) were modelled using generalized linear models to quantify screening patterns by remoteness and area-level disadvantage. Bayesian spatial models were used to obtain smoothed estimates of participation across 2,247 small areas during 2019–2020 compared to the national average, and during 2015–2016 and 2017–2018 for comparison. Spatial heterogeneity was assessed using the maximized excess events test.

**Results:**

Overall, screening participation rates was around 44% over the three time-periods. Participation was consistently lower in remote or disadvantaged areas, although heterogeneity was evident within these broad categories. There was strong evidence of spatial differences in participation over all three periods, with little change in patterns between time periods. If the spatial variation was reduced (so low participation areas were increased to the 80th centile), an extra 250,000 screens (4% of total) would have been conducted during 2019–2020.

**Conclusions:**

Despite having a well-structured evidence-based government funded national bowel cancer screening program, the substantial spatial variation in participation rates highlights the importance of accounting for the unique characteristics of specific geographical regions and their inhabitants. Identifying the reasons for geographical disparities could inform interventions to achieve more equitable access and a higher overall bowel screening uptake.

## Introduction

Colorectal cancer (bowel cancer) is the third most frequently diagnosed cancer worldwide and the second leading cause of cancer death with an estimated 935,000 such deaths globally in 2020 [1]. In Australia, there were 15,540 new cases diagnosed in 2021, making colorectal cancer the fourth most diagnosed cancer in the country and the second leading cause of cancer deaths among Australians with 5,295 deaths due to colorectal cancer in 2021 [2].

Screening has been proven to reduce both colorectal cancer incidence and mortality through the early detection and treatment of pre-cancerous lesions and early-stage cancer [3, 4] and organized bowel cancer screening programs are in place in many developed nations [5]. In Australia, the government-funded National Bowel Cancer Screening Program (NBCSP) offers free biennial bowel cancer screening, through immunochemical fecal occult blood test (iFOBT), to eligible Australian residents aged between 50 and 74 years [6]. Despite the known health benefits, [7] only 44% of eligible Australians participated in the NBCSP during 2019–2020 with slightly higher rates among females (46%) than males (42%) [6]. This low participation is consistent with that observed in many other developed countries [5, 8].

Internationally, colorectal screening participation rates have been associated with various contextual factors including socio-economic status, ethnicity, and rurality [8–13]. Geographical variation in NBCSP screening participation has been previously reported, with lower uptake in remote or more disadvantaged areas, [6, 14] and variations between larger administrative areas (range population: 0 to 23,2034 people, average: 56,483 people) across Australia [15]. However, patterns in these relatively coarse geographical structures are aggregations of heterogenous sub-populations and even locations, thus the ability to uncover area-level factors associated with screening participation has to date been limited.

Small-area mapping can help identify localized variation in colorectal cancer screening that may be masked by estimates for area structures with coarser granularity and guide education and enhanced screening efforts in areas of need [16, 17]. Here publicly available administrative data [18] was used to quantify and describe small area spatial patterns in population participation rates in the NBCSP.

## Methods

### Ethics

No specific ethical approval was required for this study as it was based on the analysis of publicly available administrative health data.

### Data

Geographical area was defined by Statistical Area 2 (SA2) from the 2016 Australian Statistical Geography Standard [19]. In 2016 SA2s had a population of between 0 and 40,181 people (average: 9,052 people). In total, 2,247 SA2s were included in the modelling, after excluding remote islands and SA2s with small populations (<5 residents annually, on average). Area-disadvantage was measured using the 2016 census-based Index for Relative Socioeconomic Advantage and Disadvantage (IRSAD [20] and remoteness defined using the 2016 Remoteness Areas [21] classification.

The NBCSP involves a screening test kit being mailed to eligible Australians, which must be completed and returned to a laboratory for analysis within 6 months of receiving the kit [6]. Publicly available quarterly data on participation in the NBCSP for eligible Australians residents aged 50–74 (further details in S1 Appendix) was obtained from the Australian Institute of Health and Welfare (AIHW) [18]. Geographical data on participation was only available for persons by residential SA2 for the combined 50–74 years age band. In 2015 the NBCSP was restricted to Australians aged 50, 55, 60, 65 and 70 years, and then progressively expanded to all Australians between the ages of 50 and 74 by 2020 [6]. People with unknown SA2’s or SA2’s for which data was suppressed by the AIHW were excluded, corresponding to <0.5% of all screened people for each time-period (S1 Fig).

### Statistical analysis

Bayesian spatial models were implemented in R version 4.2.1 [22] with the CARBayes package (version 5.2.5) [23]. All other statistical analyses were conducted using Stata/SE Version 17.0, (RRID:SCR_012763).

#### Participation by broad geographical areas

Variations in screening participation rates across broad geographical groupings of state/territory, remoteness and area disadvantage were quantified using the adjusted indirectly age standardised participation rate ratio (SPRR) estimated from multivariable negative binomial generalized linear models [24]. The log of the expected counts was included as the offset to adjust for population size and age distribution. Covariates were area-disadvantage, remoteness, and state/territory. Negative binomial models were chosen to account for overdispersion in the data as likelihood ratio tests found these models resulted in a better fit than Poisson models (see further details in S1 Appendix). Exponentiated coefficients from the models are presented as participation rate ratios (PRR) with 95% confidence intervals (CI). Wald tests were used to assess the statistical significance of individual coefficients and interaction terms (significant if p < 0.05, two-sided). Marginal participation rate ratios (SPRR) stratified by area-level factors were also estimated.

#### Participation by SA2 and time-period

For each SA2, participation was quantified using the indirectly age-standardised Participation Ratio (SPR), which is the ratio of the observed versus expected counts of screened people by SA2 and time-period. Expected counts were calculated using national age- and period-specific screening rates and incorporated the varying age eligibility of the different time periods (see S1 Appendix).

#### Bayesian spatial models

Bayesian spatial models were used to smooth area-level participation rates, protect confidentiality, and distinguish spatial patterns from the unstable observed counts, as described previously [25]. Briefly, the observed counts were modelled as a Poisson process, offset by the expected counts which were based on the national age-specific rates. This specification resulted in smoothed, indirectly age-standardised participation rate ratios that describe small area participation rates relative to the national average rate. A Leroux prior [26] incorporated the spatial dependency between adjacent areas, so that each area’s standardised participation rates were Gaussian with mean proportional to the average standardised participation rates of its neighbors. The estimates of the smoothed SPR (sSPR) and smoothed counts were taken to be the median value of the posterior distribution for each SA2 generated by the Bayesian model (further details in S1 Appendix).

All maps in this manuscript and the Supplementary Information were created in-house by first author (PD) using public domain mapping data and plotting software. Digital geographical boundary files are publicly available from the Australian Bureau of Statistics [19] for use without license and maps were created using ggplot2 version 3.3.6 [27] and R version 4.2.1. [22]. R code for generating maps has been included in the Supplementary Information (S2 Appendix).

The SA2-specific sSPR was mapped using a diverging color gradient, with pale yellow (cream) representing the national average for the same time-period, orange/red shades higher than average and blue lower than average participation (grey indicates excluded areas with small populations). The color gradient was linear on the log scale.

The posterior probability (PP) that the sSPR was greater than one [28, 29] for each small area were calculated to provide evidence that the participation rates were different to the national average (further derails in S1 Appendix). Results were presented as maps in which green represented low PP values (<0.2) and suggested that screening participation rates were truly below average, conversely purple represented high PP (>0.8) indicating participation rates were truly above average. Values between 0.2 and 0.8 (pale grey) suggested a lack of evidence of a difference from the national average [29].

Maps of the smoothed counts of screened persons used cream shades to indicate SA2-specific smoothed counts of three or less, orange shades (counts of 1000–2000) and magenta (smoothed counts >2000).

Finally, the number of missed bowel screens that could be attributed to variations in NBCSP participation rates during 2019–2020 was quantified by comparing screening rates to the top 20^th^ centile of ranked SA2s (further details in S1 Appendix).

Summary box plots were used to show the distribution of the modelled estimates by broad geographical categories. Evidence for spatial variation across Australia for each time-period, was assessed using Tango’s Maximized Excess Events Test, which compares the modelled number of screens with the expected count of screens based on the Australian average [30].

## Results

### Overall

Participation in the NBCSP increased from 1,294,442 people in 2015–2016 to 2,522,177 people during 2019–2020 (Table 1). The number of eligible invitees also increased over time from around 3 million (2015–2016) to 6 million (2019–2020), mainly due to the increasing number of ages eligible for screening across the three time-periods (Table 2). A slight increase in the overall participation rates was observed from 40.9% (2015–16) to 43.8% (2019–2020) (Table 1).

**Table 1:** Demographic characteristics by time-period for eligible persons screened through the National Bowel Cancer Screening Program, Australia, 50–74 years, 2015–2020.

	2015–2016^a^			2017–2018^a^			2019–2020		
Variable	Invitees^b^	Screened^c^	Participation Rate (%)^d^	Invitees^b^	Screened^c^	Participation Rate (%)^d^	Invitees^b^	Screened^c^	Participation Rate (%)^d^
State/territory									
New South Wales	1,034,012	394,942	38.2	1,645,025	647,242	39.3	1,869,033	778,347	41.6
Victoria	792,634	331,962	41.9	1,261,636	569,868	45.2	1,448,103	673,408	46.5
Queensland	623,375	252,307	40.5	1,011,186	412,972	40.8	1,145,808	471,583	41.2
South Australia	240,393	113,045	47.0	380,599	180,958	47.5	432,608	209,543	48.4
Western Australia	322,950	138,660	42.9	521,537	228,650	43.8	586,428	268,775	45.8
Tasmania	78,778	36,507	46.3	125,073	59,753	47.8	141,033	68,019	48.2
Northern Territory	20,200	5,996	29.7	37,783	11,104	29.4	37,869	10,176	26.9
Australian Capital Territory	48,148	21,023	43.7	76,248	34,231	44.9	90,938	42,326	46.5
Area-level disadvantage^e^									
Most advantaged	668,974	284,227	42.5	1,062,445	473,117	44.5	1,184,880	556,560	47.0
Q4	607,641	251,846	41.4	972,249	420,694	43.3	1,125,003	506,511	45.0
Q3	648,775	265,897	41.0	1,042,838	442,379	42.4	1,205,588	527,298	43.7
Q2	642,776	265,928	41.4	1,026,609	436,707	42.5	1,162,598	506,318	43.6
Most disadvantaged	591,700	226,269	38.2	952,663	370,904	38.9	1,070,241	423,995	39.6
Remoteness^f^									
Major cities	2,164,677	863,851	39.9	3,449,056	1,436,070	41.6	3,971,707	1,722,590	43.4
Inner regional	650,459	289,105	44.4	1,046,458	476,202	45.5	1,172,420	547,146	46.7
Outer regional	300,786	125,887	41.9	486,078	206,031	42.4	526,069	225,004	42.8
Remote	44,568	15,599	35.0	77,495	26,475	34.2	81,624	27,437	33.6
Total	3,160,490	**1,294,442**	**40.9**	5,059,087	**2,144,778**	**42.4**	5,751,820	**2,522,177**	**43.8**

^a.^ Each 2-year period covered the calendar year from 1 January in the first year to 31 December in the following year.

^b.^ Number of eligible people invited to screen for each time-period.

^c.^ Number of invitees who returned a completed bowel screening test kit within each time-period or up to 6 months thereafter.

^d.^ Number of screened people divided by the number of invitees, expressed as a percentage.

^e.^ Area-level disadvantage was defined by the 2016 SEIFA Index of Relative Socioeconomic Advantage and Disadvantage.

^f.^ Remote areas were defined by the Remoteness Areas 2016 classification with remote and very remote areas combined.

**Table 2:** Eligible ages invited for screening by time-period, National Bowel Cancer Screening Program, Australia 2015–2020.

Period of invitation for screening	Ages invited during that period
1 January 2015–31 December 2015	50, 55, 60, 65, 70,74
1 January 2016–31 December 2016	50, 55, 60, 64, 65, 70, 72, 74
1 January 2017–31 December 2017	50, 54, 55, 58, 60, 64, 68,70, 72, 74
1 January 2018–31 December 2018	50, 54, 58, 60, 62, 64, 66, 68, 70, 72, 74
1 January 2019–31 December 2020	50, 52, 54, 56, 58, 60, 62, 64, 66, 68, 70, 72, 74

### Broad geographical patterns

**Fig 1:**
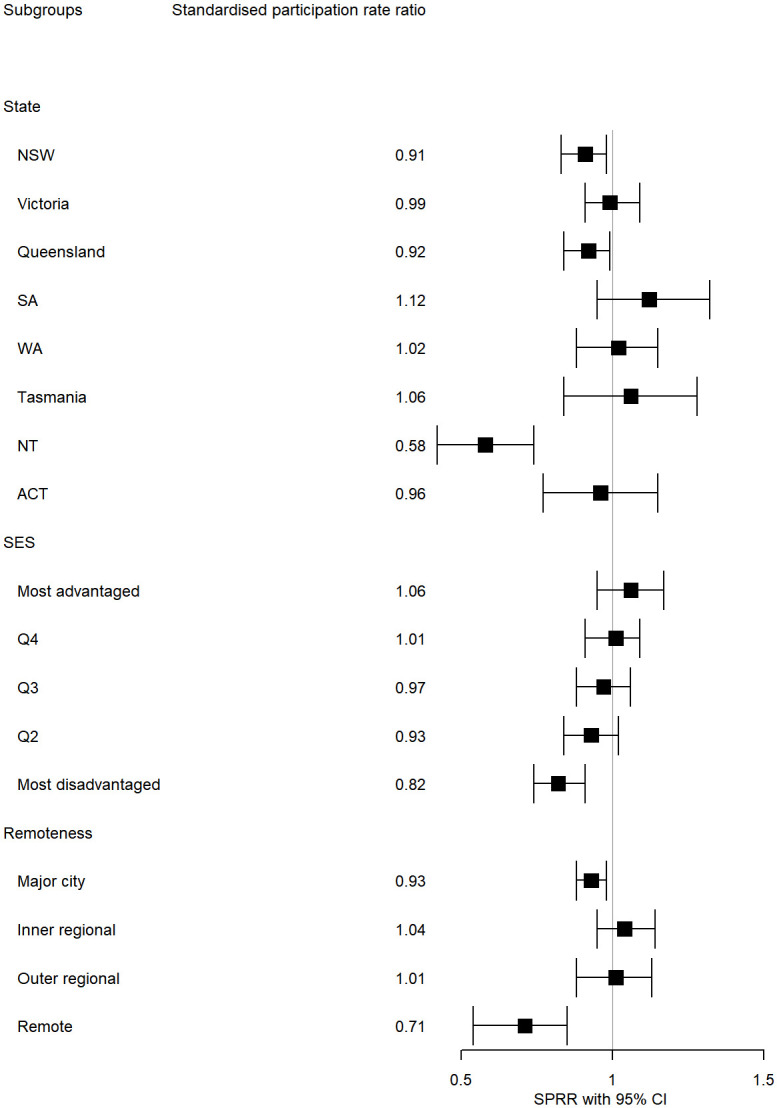
Plot of adjusted standardised participation rate ratios (SPRR) by state*/* territory, area-level socio-economic quintile and remoteness category, with 95% confidence intervals., Australia, 2019–2020. Abbreviations for states/territories are NSW: New South Wales, WA: Western Australia, SA: South Australia, ACT: Australian Capital Territory, NT Northern Territory.

During 2019 to 2020, after adjustment for state/territory, remoteness and area disadvantage, screening participation was lower among people living in the most socioeconomically disadvantaged areas compared to the most advantaged areas (S1 Table). Moreover, remote areas had lower participation whereas inner regional areas had higher participation than major cities (Fig 1, S1 Table). Patterns were similar for the other two time-periods.

### Spatial patterns

**Fig 2:**
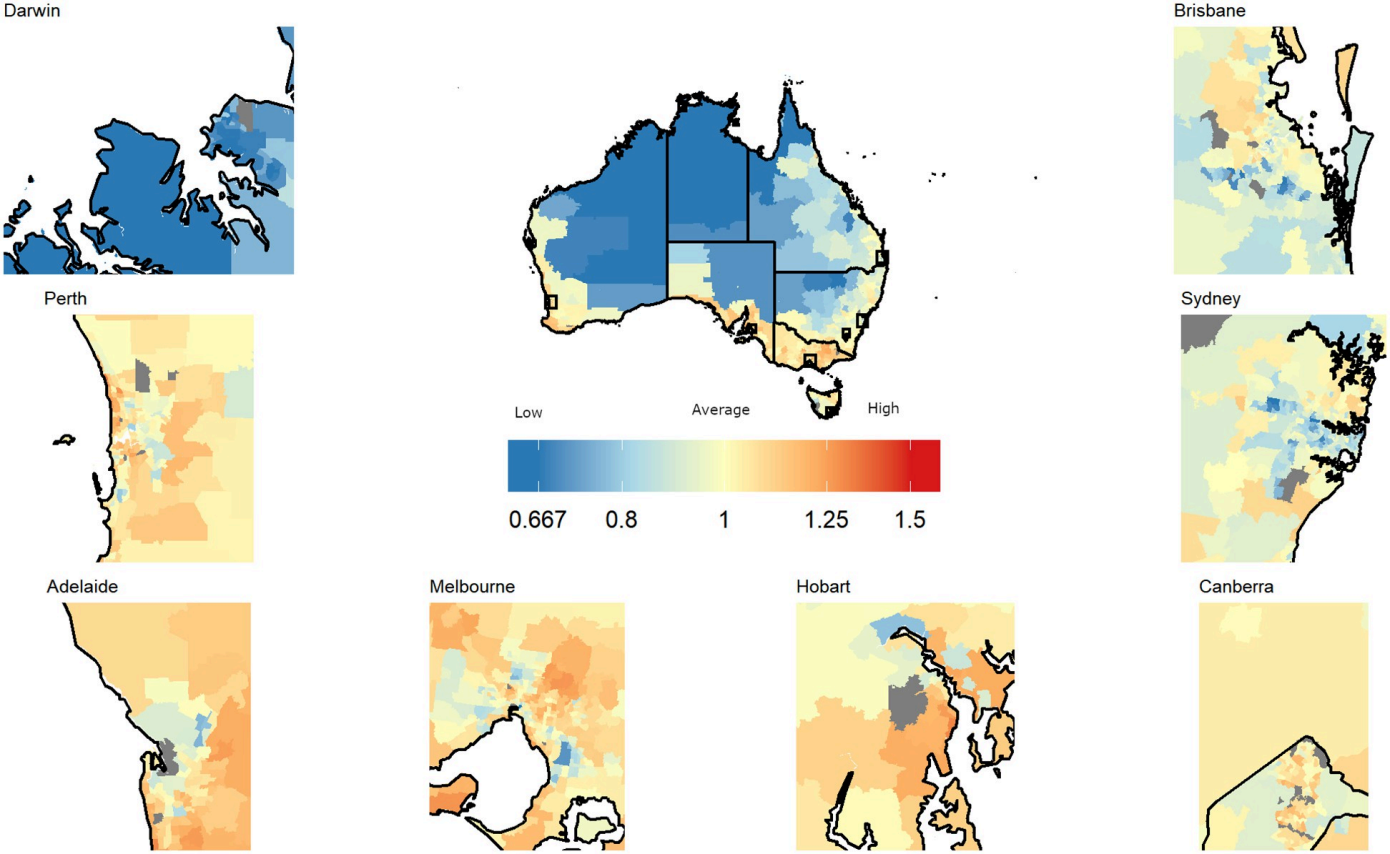
Maps of the smoothed standardised participation ratios (sSPRs) for bowel cancer screening by Statistical Area Level 2, persons, Australia, 2019–2020, with insets of the state and territory capitals. The map for Canberra includes the boundary between the Australian Capital Territory and New South Wales. An SPR with value 1 indicates that screening participation is the same as the national average (43.8%) over 2019–2020. Grey indicates areas not included in spatial analysis due to small populations.

There was strong evidence of spatial variation in screening participation for all three time periods (all maximized excess events test: p<0.001). Maps of the smoothed SPR estimates for 2019 to 2020 (Fig 2), 2015–2016 (S2 Fig) and 2017–2018 (S3 Fig) showed that participation was consistently lower than the national average in the Northern Territory, north Queensland and inner regions of Western Australia and South Australia (blue shades). In contrast, rates were consistently higher than average for coastal areas of Victoria, South Australia, Western Australia and Tasmania (orange/red shades). Finally, many regions along the south-eastern coast of mainland Australia (cream-colored) had similar participation to the national average.

**Fig 3:**
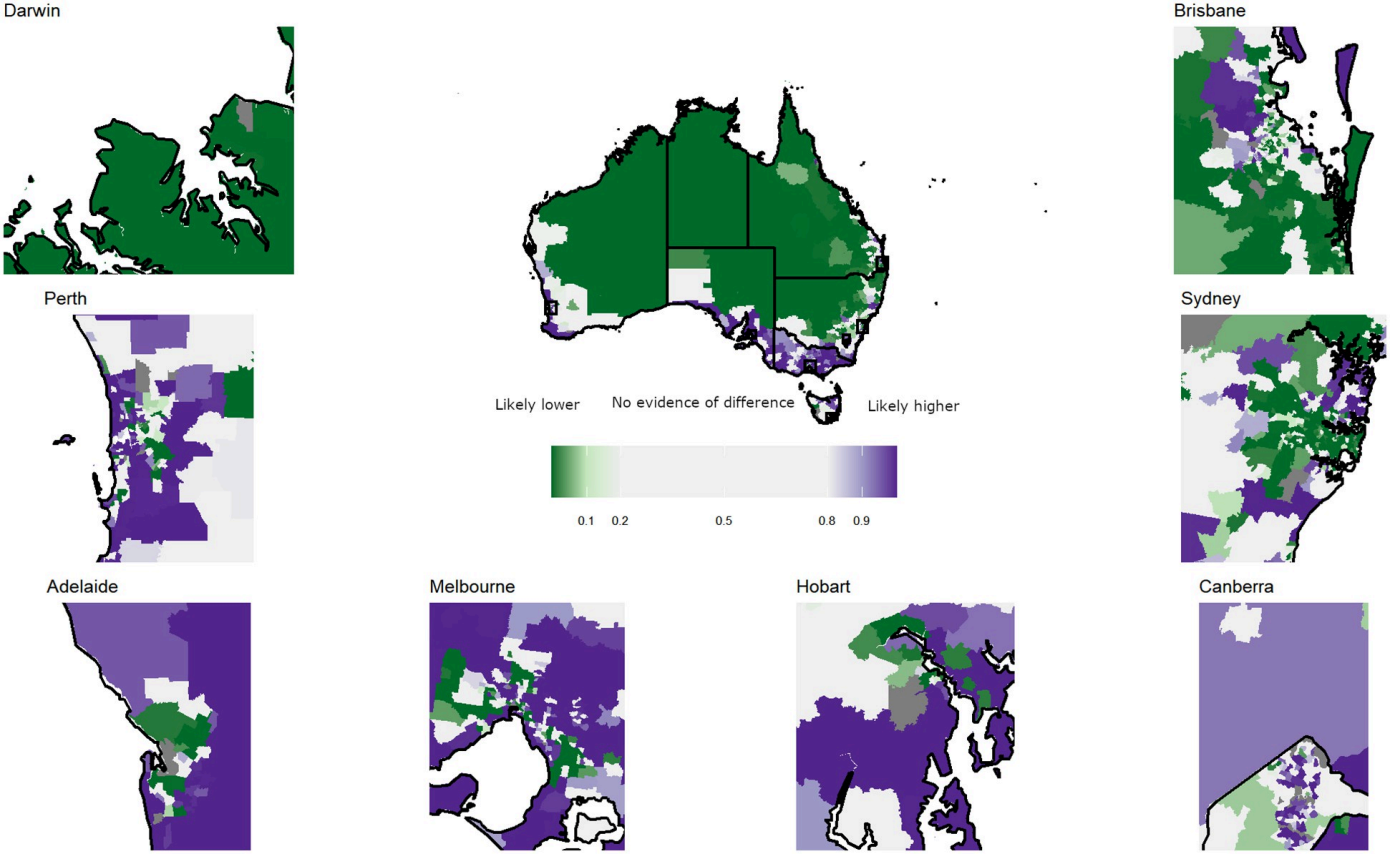
Maps of posterior probability (PP) for bowel cancer screening by Statistical Area Level 2 and time-period, persons, Australia., 2019–2020 with insets for selected major state and territory capitals. Values of the PP for smoothed standardised participation ratios are truly lower than average (PP <0.2), uncertain (PP = 0.2–0.8) and higher than average (PP >0.8). The map for Canberra includes the boundary between the Australian Capital Territory and New South Wales.

Based on the posterior probabilities (Fig 3), there was evidence that during 2019–2020, 807 (36%) small areas (out of 2,247) had participation rates that were likely to be lower than the national average (probability of <20% that the sSPR was greater than 1). Rates were higher (probability of >80% that the sSPR was greater than 1) than the national average for 1,106 small areas.

Patterns for capital cities were generally consistent with those for their respective states/territories. For example, there was evidence that rates for areas of Darwin were consistently lower than average (as for the Northern Territory) whereas rates were likely to be higher than average in Adelaide over all time-periods (Fig 3, S4 and S5 Figs).

**Fig 4:**
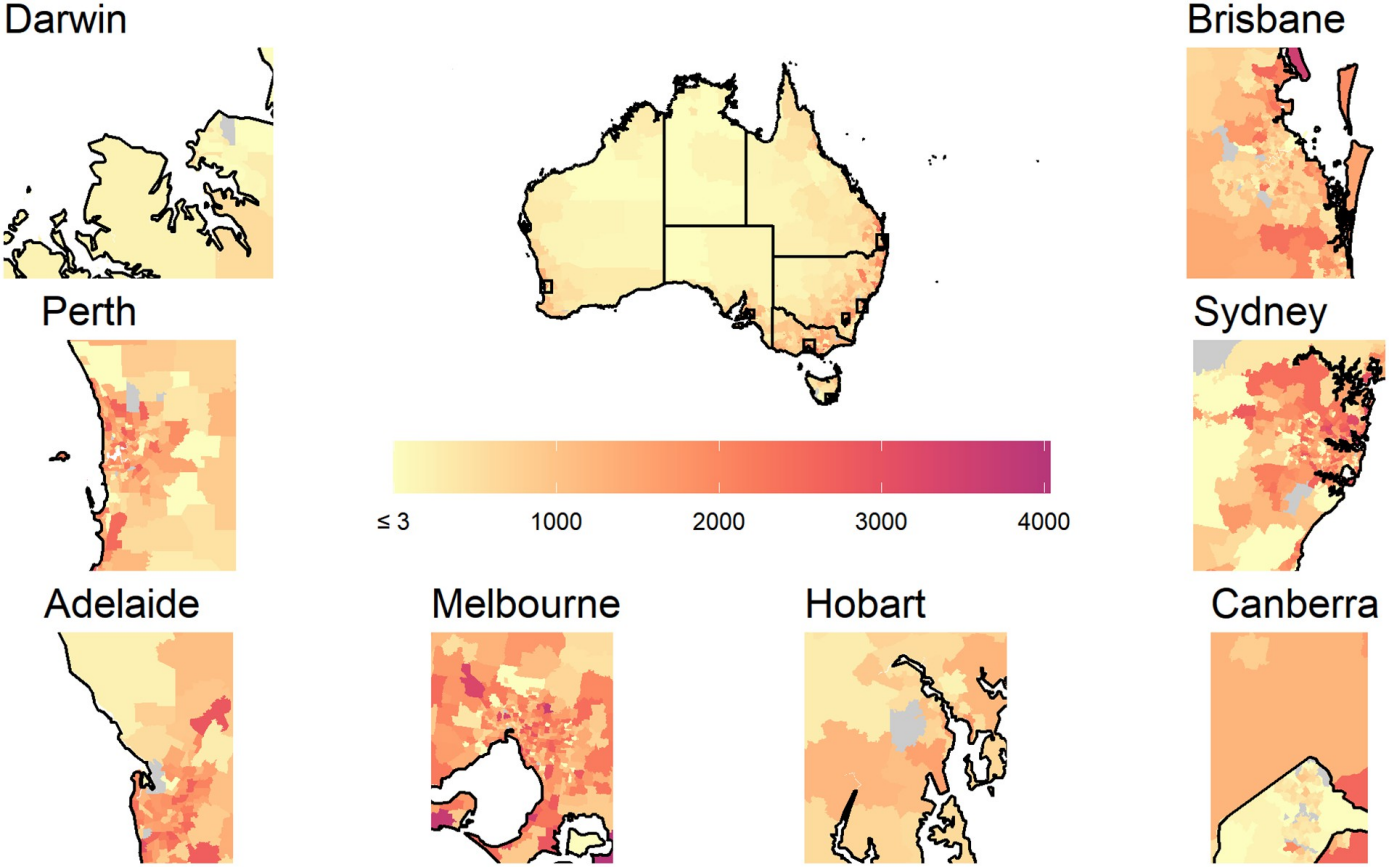
Smoothed counts of participants in National Bowel Cancer Screening Program, Australia., 2019–2020 with insets for selected major state and territory capitals. Counts below 3 are truncated at 3. The map for Canberra includes the boundary between the Australian Capital Territory and New South Wales.

While the national map showing smoothed counts of screened persons (Fig 4) was dominated by areas with three or fewer counts (cream shades), this reflects the lower populations in the geographically larger but sparsely populated rural and remote areas of Australia. Most areas with larger smoothed counts in darker shades of orange and magenta were in the more densely populated coastal areas, especially in and around the capital cities.

The distribution of the median smoothed SPR varied by remoteness categories during 2019–2020, with lower participation than average for remote areas compared to major cities and regional areas where corresponding estimates tended to be around the average (S6A Fig). There was no evidence that these patterns varied by area disadvantage with the spread of the estimates being similar (S6B Fig). Estimates were consistently lower than average for the Northern Territory (S6C Fig). Patterns did not vary by time-period.

By using the benchmark of the participation rates in the top 20^th^ centile of SA2s across Australia, we estimated that about 254,201 additional screens would have been carried out during 2019–2020 if the screening participation in the remaining 80% of SA2’s had been equal to that of the top 20^th^ centile (S2 Table). By doing this, the national screening rate over all SA2’s during 2019–2020 would have increased to about 48%.

For additional context, supplemental information shows the estimated resident population (ERP) for persons aged 50 to 74 years by 2016 SA2’s (S7 Fig), estimated percentage of the ERP who were not invited to screen nationally by SA2 for 2019–2020 (S8 Fig) and the total ERP for each of the major capital cities in Australia (S3 Table).

## Discussion

Despite having a well-structured bowel cancer screening program supported by government funding and grounded in scientific evidence, our study provides compelling evidence of spatial discrepancies in bowel screening participation rates across Australia. It also highlighted the variability within demographic groups, demonstrating the importance of geographic analyses of screening participation rates at this finer granularity. We estimated that the spatial variation in NBCSP participation resulted in over 250,000 missed screens during 2019–2020.

Limitations include potential geographical misclassification as SA2 boundaries were mapped using published population-weighted geographic correspondences that were based on entire populations, and hence may not be representative of specific age groups. We were also unable to account for the impact of other likely confounders such as sex, First Nations status and ethnicity, [6, 14] hence our ability to assess potential drivers of the observed patterns was limited. Rates in some areas may be influenced by screening outside the NBCSP.

Our findings are consistent with previous studies that reported lower bowel screening uptake among more disadvantaged or remote areas in Australia [6, 14, 31] and globally. [8, 12, 32] That these patterns were evident across three non-overlapping consecutive time-periods indicates the stability of these associations. A unique feature of this study was the ability to examine how screening participation varied not only between but also within these broader categories (S6 Fig). For example, there was substantial heterogeneity within nearly all area-disadvantage and remoteness categories except for the mostly lower than average rates within remote SA2s. This suggests that while participation varied by these broad geographical classifications, there are other unidentified factors either on an individual or area-level influencing screening participation within these regions. Relying on broad geographical categories may not capture important variability in screening within these entities. Further analyses to identify these factors are warranted.

The drivers of low bowel screening participation are likely to be complex and multifaceted [33, 34]. Geographical and socio-demographic disparities in cancer screening are not unique to colorectal cancer screening; for example residents of remote or disadvantaged areas have lower participation rates in all three of Australia’s population-based screening programs (breast, colorectal, cervical) [7] and similar inequities have also been observed internationally [9, 11, 12, 35]. While these disparities likely reflect a range of contextual factors that are common to all cancer screening activities, including reluctance to engage in any cancer screening activity and perceived relevance of screening activity, [36] additional barriers related to the iFOBT test procedure itself, including perceived unpleasantness and discomfort with the process may further reduce bowel screening participation [33, 34].

In rural areas, bowel screening in particular is likely to be impacted by poorer access to postal facilities that are crucial for distributing and returning screening kits, poorer access to digital media [37] which are being increasingly used to promote the NBCSP, [38] or even characteristics of rural communities themselves that affect health seeking behaviors generally [39]. Rural Australians often have poorer access to health care [40] and are less likely to engage regularly with primary care providers, [41] who are instrumental in encouraging participation in bowel screening [42]. In addition, the validity of iFOBT kits is affected by heat [43] and many remote areas in Australia experience extreme temperatures for a large proportion of the year. The NBCSP has a “Hot Zone Policy”, so that screening invitations are sent to residents of certain areas of the Northern Territory, North Queensland and Western Australia during the cooler months [44]. In these areas, recipients must ensure that the kit is posted in the evening, preferably at a post office, to maintain sample integrity. This is logistically impossible for many remote Australians. Local health professionals in some remote communities encourage and facilitate bowel screening outside the NBCSP through medical clinics [45] meaning our results may underestimate screening behavior in these areas. Lower screening rates in remote areas may also have been impacted by higher levels of disadvantage such as lower education and poorer health literacy among rural populations [46] which can affect screening participation [14, 47].

Commonly cited reasons for the generally lower screening participation among people from more disadvantaged areas include differences in knowledge and attitudes, reduced responsiveness to disease prevention and health promotion messages and a lower engagement in healthy behaviors in general [8, 14, 31, 33]. However, our results found that NBCSP participation rate in some areas categorized as most disadvantaged is higher than the national average, demonstrating that area-level disadvantage lacks sufficient specificity for identifying areas with poorer participation. Therefore, for more effective and efficient initiatives for improving participation rates, it is imperative to understand what is influencing the heterogeneity within these socio-economic classifications. This heterogeneity provides an opportunity for areas with poor participation rates to learn from areas of similar socio-economic characteristics and similar access to health services but higher screening uptake.

Socio-economic disadvantage is typically higher in more remote areas of Australia. Nevertheless, after adjustment for area-level socio-economic disadvantage, there has been a consistent pattern for remoteness to remain independently associated with geographical variations in colorectal cancer screening [9, 14, 48]. Consistent with these studies, when looking at the broad geographical patterns, our study found that the significant association between remoteness and colorectal cancer screening remained even after adjusting for area-level disadvantage, suggesting that factors in addition to socio-economic status are important in explaining the pattern of lower screening rates in more remote areas of Australia. As such, rather than considering these two geographical measures separately, the interaction between remoteness and area-level socio-economic disadvantage needs to be considered in terms of their respective impacts on screening patterns.

Similar to cervical screening, [49] First Nations Australians have substantially lower bowel screening rates than other Australians [6]. It is possible that at least some portion of the lower participation in more remote regions may reflect the higher proportions of First Nations people living in these areas [50]. Historically, First Nations Australians have faced additional barriers to screening such as the lack of culturally appropriate information on colorectal cancer risk, screening benefits and iFOBT kit usage [51, 52]. However, various initiatives to improve bowel screening uptake among First Nations Australians are being implemented, including developing culturally appropriate information [53] and trialling alternate methods of distributing the screening kits [52]. Since late 2022, these methods are being phased into the NBCSP [54].

We lacked information on the number of people who opted-out of participation in the NBCSP by small areas, or their reasons for doing so. Valid medical reasons for opting out include a recent or scheduled colonoscopy or other form of bowel screening [55]. However, the extent of screening outside the NBCSP is unknown. Based on NBCSP opt-out data between 2006 and 2017, over a third of people who opted out of the NBCSP were screened outside the program [55]. We estimated that around 2% of the six million people invited to screen during 2019–2020 may have been screened outside the program. However, given the percentage who opt-out is only a small percentage of the eligible population for screening, and many areas had participation rates 20–35% lower than the national average, our results highlight the current inequalities in NBCSP screening, particularly among people living in socioeconomically disadvantaged areas.

Given the cost-effectiveness of non-invasive iFOBTs versus invasive colonoscopies and similar efficacy in average-risk populations, [56] if the full benefits of the NBCSP are to be realized it is vital that the current NBCSP uptake is increased [4, 31]. A recent study suggested that the NBCSP could potentially save around 76,000 lives over the period 2015–40 if coverage rates were increased to 50% [4]. We have shown that, by increasing participation in all areas to the benchmark of the top 20^th^ centile of SA2s, the national participation rate would increase to around 48%. Based on the previously mentioned study, this increase would result in thousands of lives being saved and highlights the important of identifying the reasons for these spatial patterns. In an equitable country, health outcomes should not depend on where people live.

Identifying factors driving bowel screening participation at the area level through ecological or multilevel analyses could help inform the development of potential interventions at the more localised level. While outside the scope of this study, further work could compare individual-level factors associated with screening participation in areas with high screening activity versus those with low screening activity as has been done recently for cervical screening in Queensland, Australia [57]. Results of such an analysis could provide public health decision makers and relevant stakeholders greater insights into key drivers of bowel screening behavior within their community as well as inform targeted strategies to improve screening in lower screening areas.

Study strengths include the use of a publicly available population-based database with complete coverage of all people who were eligible for screening through the NBSCP. Complex modelling techniques were applied including Bayesian spatial smoothing methods to generate robust small area estimates of screening participation while preserving data confidentiality. To the best of our knowledge, such methods have not been previously applied to bowel screening data at the small area level either in Australia or internationally.

Given the changes over time in the eligible ages for the NBCSP, comparisons of spatial patterns across time-periods in this study should be made cautiously. Consequently, we have not included a temporal component in the models. It was not possible to disentangle the impact of changes in statistical power resulting from the increase in eligible population during 2015–2020, the temporal changes in spatial variability and temporal changes in overall participation, all of which are likely to impact observed small-area patterns.

## Conclusions

By uncovering small-area patterns in bowel cancer screening participation, this study provides a focus to motivate further research into the drivers of participation and non-participation. Such research may allow insights and provide opportunities to increase overall screening participation rates by reducing the currently observed geographical inequalities. Our results highlight that any initiatives to enhance screening participation, both within Australia and internationally, must consider the unique characteristics and necessities of specific geographical regions and their inhabitants. Given low overall participation rates in Australia, identifying why some small areas, irrespective of disadvantage and most remoteness categories, have higher than average participation rates, may be important to ensure that greater inequity does not result from attempts to increase participation. participation.

## Supporting information

S1 Appendix Supplemental methods. (PDF)

S2 Appendix Sample R code. (PDF)

S1 Dataset Estimated smoothed standardised participation ratios (SPR), modelled counts and posterior probability (PP) that the smoothed SPR was greater than one, bowel cancer screening, Australia 2015–2020. (XLSX)

S1 Table Participation rate ratios (PRR) for bowel cancer screening, from multivariable generalized Poisson models persons, Australia, 2019–2020. (PDF)

S2 Table Number of missed bowel cancer screens, Australia, 2019–2020. (PDF)

S3 Table Estimated resident population (ERP) for each capital city, Australia, 2021. (PDF)

S1 Fig Schematic diagram of selection process for screening data set. Abbreviations are SA2 (statistical area level 2), NBCSP (National Bowel Cancer Screening Program), AIHW (Australian Institute of Health and Welfare).
(TIF)

S2 Fig Maps of the smoothed standardised participation ratios (sSPRs) for bowel cancer screening for persons by Statistical Area Level 2, 2015–2016, with insets of the state and territory capitals. The map for Canberra includes the boundary between the Australian Capital Territory and New South Wales. An SPR with value 1 indicates that screening participation is the same as the national average (40.9%) during 2015–2016.
(TIF)

S3 Fig Maps of the smoothed standardised participation ratios (sSPRs) for bowel cancer screening for persons by Statistical Area Level 2, 2017–2018, with insets of the state and territory capitals. The map for Canberra includes the boundary between the Australian Capital Territory and New South Wales. An SPR with value 1 indicates that screening participation is the same as the national average (40.9%) during 2015–2016.
(TIF)

S4 Fig Maps of posterior probability (PP) for bowel cancer screening by Statistical Area Level 2 and time-period, persons, Australia., 2015–2016 with insets for selected major state and territory capitals. Values of the PP for smoothed standardised participation ratios are truly lower than average (PP <0.2), uncertain (PP = 0.2–0.8) and higher than average (PP >0.8). The map for Canberra includes the boundary between the Australian Capital Territory and New South Wales.
(TIF)

S5 Fig Maps of posterior probability (PP) for bowel cancer screening by Statistical Area Level 2 and time-period, persons, Australia., 2015–2016 with insets for selected major state and territory capitals. Values of the PP for smoothed standardised participation ratios are truly lower than average (PP <0.2), uncertain (PP = 0.2–0.8) and higher than average (PP >0.8). The map for Canberra includes the boundary between the Australian Capital Territory and New South Wales.
(TIF)

S6 Fig Distribution of smoothed median standardised participation ratios (SPRs) by remoteness (A) socio-economic status (B), and state/territory (C), for bowel cancer screening, Australia 2019–2020. The vertical line indicates an SPR of 1 representing the national average. Abbreviations are M.Adv Most advantaged, M.Disadv Most disadvantaged, NSW: New South Wales, WA: Western Australia, SA: South Australia, ACT: Australian Capital Territory, NT Northern Territory.
(TIF)

S7 Fig Estimated resident population (ERP) of persons aged 50–74 by Statistical Area Level 2 (SA2), Australia, 2016, with insets for selected major state and territory capitals. The map for Canberra includes the boundary between the Australian Capital Territory and New South Wales.
(TIF)

S8 Fig Percentage of the estimated resident population aged 50–74 years, who were not eligible to screen in National Bowel Cancer Screening Program, Australia., 2019–2020 with insets for selected major state and territory capitals. The map for Canberra includes the boundary between the Australian Capital Territory and New South Wales.
(TIF)

S1 Checklist STROBE statement—Checklist of items that should be included in reports of *cohort studies*. (DOCX)
