# Supplementary material for: Geographical and spatial variations in bowel cancer screening participation, Australia, 2015–2020
Source: PLoS One. 2023 Jul 20;18(7):e0288992. doi: 10.1371/journal.pone.0288992 (PMC10358922; doi:10.1371/journal.pone.0288992)
Supplement: S1 Appendix — (PDF) [file pone.0288992.s001.pdf]

S1 Appendix Supplemental methods

Supplemental Methods includes further details on the data set, statistical analysis and Bayesian spatial models.

Data

The quarterly data on participation in the National Bowel Cancer Screening Program (NBCSP) [1] was available in two separate datasets: (a) national participation by sex provided in five-year age bands; and (b) participation for persons only by residential Statistical Area 2 (SA2) for the combined 50-74 years age band. For both datasets the specific ages invited for screening within the age band changed each year (Table 1). For this study data from three adjacent (non-overlapping) two-year screening intervals of 2015-2016, 2017-2018 and 2019-2020 were used.

For each dataset, the following information was extracted: a) the number of eligible people invited to screen (invitees) and b) the number of eligible people who returned a completed bowel screening kit within six months of the relevant period (participants). The reported number of eligible invitees (not participants) excluded those who deferred, opted out or skipped a screening round (from November 2019 onwards due to a colonoscopy within past 2 years) without completing their screening test. For example, around 5.6% (n = 342,096) of the six million people invited to screen during 2019-2020 were excluded. [2]

Overall national participation rate (average rate) was calculated as the total percentage of eligible people invited to screen through the NBCSP in Australia during the relevant 2-year period who returned a completed screening test within that period or before 30 June of the following year. [2]

Geographical analysis

The geographic unit was the Statistical Area Level 2 (SA2) from the 2016 Australian Statistical Geography Standard (ASGS). [3] SA2s cover Australia without gap or overlap and are deemed to group together relatively homogenous sub-populations regarding socio-economic status [3] and have been previously used for cancer-related spatial mapping in Australia including the "Australian Cancer Atlas [4] and cervical screening participation. [5]

The screening data obtained from the Australian Institute of Health and Welfare (AIHW) [1] had information on the SA2 based on a person's usual residence when invited to screen in the NBCSP. From 2015 to 2017, correspondence files [1] were used to assign SA2's to NBCSP invitees using either Statistical Area Level 1 (SA1) or postcode (if SA1 details were insufficient) of usual residence. [3] From 2019 to 2020 SA2 areas were directly assigned to NBCSP invitees using longitude and latitude data. For those invitees without reliable SA2 data, SA2s were mapped with a postcode to SA2 correspondence instead. As postcodes can cross boundaries of SA2's, geographical mapping based on postcode correspondences are likely to be less accurate.

If SA1 codes and postcodes could not be attributed to a SA2, those invitees were included in an 'Unknown' group in the data tables and were excluded from analysis.

Data for 2015-2016 and 2017-2018 were defined using the 2011 ASGS [6] and so were converted to the 2016 ASGS using a published population-weighted correspondence file .[7] Persons without a valid SA2 were excluded from the spatial analyses. Small areas, for which screening information was suppressed due to concerns about data quality [1] were also not considered.

Eligible people for NBCSP

The eligibility criteria for the NBCSP include being an Australian or New Zealand citizens or permanent migrant with a current Medicare card (or registered as a Department of Veterans' Affairs customer) in the invited age bracket and a current Australian mailing address. [8] People who are conditional migrants, temporary residents, or covered by a reciprocal health care agreement are ineligible. The NBCSP identifies eligible people in the target population aged 50-74 years through Medicare data. [9]

Population data

Information on the eligible invitees by SA2 was only available for all ages in the target age group (50-74) combined for each time-period, therefore, to calculate expected participation numbers by SA2s, we needed to estimate the number of eligible invites by five-year age groups. To this end, the published Estimated Resident Population (ERP) data by five-year age group, year and SA2 (2016 ASGS) for persons in Australia between 2015 and 2020 [10] was used.

The ERP is the official measure of Australia's population based on the concept of usual residence. [11] It includes all people, regardless of nationality or citizenship, who usually live in Australia, except foreign diplomatic personnel and overseas visitors who are in Australia for less than a year or more. Published ERP data by year and SA2 (2016 ASGS) for persons in Australia between 2015 and 2020 were only available by five-year age group. [10] However, we also required the population by single year age groups since NBCSP eligibility was rolled out by single ages within each five-year age band. We assumed that the population in each single year age group was spread equally within the relevant five-year age group. This was consistent with the known single-age population proportions of the ERP for total Australia by sex. [11]

Eligible population for screening

The ERP may include people who are not eligible for the NBSCP due to not meeting the residency requirements. The number of invitees [1] were compared with the ERP to estimate the proportion of the population who were ineligible for screening, both nationally and for each SA2.

The estimated percentage of the ERP who were not invited to screen nationally by five-year age groups increased with age and ranged from around 8% (50-54) to 29% (70-74) in 2019 to 2020. A map of these percentages by SA2 for 2019 to 2020 (S8 Fig) indicated that many areas along populated south-eastern coastal areas had >27% of the ERP not eligible to screen (darker shades of blue).

The distribution of areas with higher percentage of non-eligible residents (high = ≥ 27% residents were non-eligible) varied by state/territory. For example, around half of all areas in the Northern Territory and around a third in Queensland and Tasmania had a high percentage of non-eligible residents by contrast to around 9% for Victoria. The proportion of ‘high’ areas increased with remoteness (9% for major cities to 43% for remote areas) and was higher for the most disadvantaged areas (20%) than the most advantaged areas (16%).

Participation rates

Participation rates were defined as the percentage of eligible people invited to screen through the NBCSP during the relevant 2-year period who returned a completed screening test within that period or by 30 June of the following year. [2] Therefore, people invited to screen in 2015 to 2016 who

returned their completed tests in first half of 2017 were included in the numerator for 2015 to 2016 whereas those who were invited to screen during 2017 to 2018 and who returned their completed tests in first half of 2017 were included in the numerator for 2017-2018.

Expected participation counts by SA2.

The expected participation counts in each SA2(i) were calculated by multiplying the age-specific national participation rate (per 100 people, $\text{Rate}_{Australiakt}$) by the SA2's age-specific eligible population, (Invitees_{ikt}) for each five-year age group k (50-54, 55-59, 60-64, 65-69, 70-74) in each time-period, t.

$$Expected_{it} = (\sum_{k}^{K} Rate_{Australia_{kt}} \times Invitees_{ikt})/100$$

Equation A 1

The calculation of each component in Equation A 1 is described below.

Age-specific national participation rate

The age-specific national participation rate ($\text{Rate}_{Australiakt}$) for each five-year age group k and time-period t was defined as the ratio of the total number of participants in each single-year age group, s, ($\text{Count}_{Australiast}$) to the total number of eligible people invited to screen (invitees) in the same period and single year age group:

$$Rate_{Australiakt} = \left(\sum_{s \in k} Count_{Australiast} \,/\, \sum_{s \in k} Invitees_{Australiast}\right) \times 100$$

Equation A 2

The notation s ε k is read as "s in k". For example, if k is 50-54, s can take the values 50, 51, 52, 53, and 54.

Number of eligible people invited to screen by SA2

Because of the assumptions made, there were some discrepancies between the published number of people invited in each area and $\sum_k$ Elig_Pop_{kt}. To correct for this and obtain the number of people invited to screen used in Equation A 1, the preliminary estimates of the eligible population

($Elig\_Pop_{ikt}$) were adjusted. This gave us the number of eligible people invited to screen (invitees) ($Invitees_{ikt}$) for each five-year age group.

$$Invitees_{ikt} = Elig\_Pop_{ikt} \ X \ Adjust_{it}$$

Equation A 3

The adjustment factor ($Adjust_{it}$) was the ratio of the published number of invitees ($AIHW\_Invitees_{it}$) aged 50-74 years and the sum of the eligible population ($Elig\_Pop_{ikt}$) over all age groups for each SA2 and time-period. Multiplying the adjustment factor ($Adjust_{it}$) by 100 gave an estimate of the percentage of the ERP invited to screen by SA2.

$$Adjust_{it} = AIHW\_Invitees_{it} \ / \sum_{k}^{K} Elig\_Pop_{ikt}$$

Equation A 4

Eligible screening population by SA2

To obtain age-adjusted standardised rates, the age-specific population eligible for screening by SA2 was required. The SA2-level population eligible for screening is publicly available, [1] however not by age group. The eligible population differs from the age-specific ERP because of the people who do not meet the residency requirements for screening. Ineligibility due to residency status is not available at the SA2 level and so it was assumed that the proportion of people in each age group and for each period who were eligible for screening was constant across the country.

The population eligible for screening in each SA2 ($Elig\_Pop_{ikt}$) was therefore calculated by multiplying the national proportion of the age group-specific population that were invited to screen ($Prop\_invitees_{kt}$) by the sum of the population (ERP_{its}) for each five-year age group *k* where *i* is the SA2 area, *t* is the 2-year time-period, and *s* is the single year of age. An indicator variable, ($Eligible_{st}$), was used to indicate whether a specific single-year age group was eligible in each period.

$$Elig\_Pop_{ikt} = Prop\_invitees_{kt} \sum_{s \in k} Eligible_{st} \times ERP_{its}$$

Equation A 5

Note that $Eligible_{st}$ was a binary variable, where:

$$Eligible_{st} = 0 \; if \; the \; single - year \; age \; group \; was \; ineligible \; for \; screening \; at \; time \; period \; t, and$$

$$Eligible_{st} = 1 \; if \; the \; single - year \; age \; group \; was \; eligible \; for \; screening \; at \; time \; period \; t$$

For example, for 2019 to 2020, in the 50-54 age group 50-, 52- and 54-year-olds were eligible for screening. Hence, the eligible screening population for area *i* in that period was:

$$Elig\_Pop_{i,50-54,2019-20} = (Pop_{i,50,2019} + Pop_{i,50,2020} + Pop_{i,52,2019} + Pop_{i,52,2020} + Pop_{i,54,2019} + Pop_{i,54,2020}) \text{ x } Prop\_invitees_{50-54,2019-20}$$

Equation A 6

National age-specific proportion of people invited to screen

The national age-specific proportion of people invited to screen ($Prop\_invitees_{kt}$) used in Equation A 5 was the ratio of the number of invitees for Australia ($Invitees_{Australiakt}$) [1] to the eligible population for Australia ($Elig\_Pop_{Australiaikt}$) by age group and time-period where the population by single year age was ($ERP_{Australiast}$).

$$Prop\_invitees_{kt} = Invitees_{Australiakt} \; / \; Elig\_Pop_{Australiakt}$$

$$Elig\_Pop_{Australiakt} = \sum_{s \in k} Eligible_{st} \times ERP_{Australiast}$$

$$Eligible_{st} = 0 \; if \; the \; single \; year \; of \; age \; was \; ineligible \; for \; screening \; at \; time \; period \; t$$

$$Eligible_{st} = 1 \; if \; the \; single \; year \; of \; age \; was \; eligible \; for \; screening \; in \; time \; period \; t$$

Equation A 7

Generalized linear models.

Variations in screening participation rates across broad geographical groupings were explored using multivariable negative binomial generalized linear models. [12] The observed number of screens was modelled with the log of the expected counts included as the offset to adjust for population size and age distribution. Covariates were area-disadvantage, remoteness, and state/territory. Area-disadvantage was measured by the 2016 census-based Index for Relative Socioeconomic Advantage and Disadvantage (IRSAD), [13] and remoteness defined using the 2016 Remoteness Areas classification, [14] with remote and very remote categories combined.

Separate models were fitted for each time-period. Negative binomial models were chosen to account for overdispersion in the data as likelihood ratio tests found these models resulted in a better fit than the Poisson distribution.

A backwards stepwise model building process was used, with variables retained based on likelihood ratio tests ($p<0.20$). Starting with the fully adjusted main effects model, interaction terms between different covariate pairs were also tested. No interactions were included in the final model since their inclusion did not improve the model fit ($p \geq 0.20$).

Exponentiated coefficients from the models were reported as participation rate ratios (PRR) with 95% confidence intervals (CI). Wald tests were used to assess the statistical significance of individual coefficients and interaction terms (significant if $p < 0.05$, two-sided). Marginal participation rate ratios stratified by area-level factors were also estimated.

Bayesian spatial model for screening participation

The Bayesian spatial models used to quantify small-area spatial patterns in screening participation is given by Equation A 8, where γ_i is the observed count of screened people in area *i*, E_i the expected number of counts (calculated above as $Expected_{it}$), θ_i the log indirectly age-standardised Participation Ratio (SPR), β_0 the overall fixed effect (intercept), and *InverseGamma* refers to the inverse gamma distribution with parameters shape and scale. S_i is the spatial random effect modelled with the Leroux prior [15]: where $\mathbf{S}_{\backslash i}$ is the sum of the neighbouring random effects (with weight ρ indicating the proportion of the area effects that is spatially correlated) and w_{ij} the $(i,j)^{th}$ entry of the pairwise

adjacency matrix (w_{ij} = 1 if areas *i* and *j* are adjacent, 0 otherwise). Modelled estimates are effectively age-adjusted since E_i considers the age structure and eligible population size. These models modelled the log of the Poisson rate, with a baseline value and a spatial term and no covariates.

$$y_i \sim Poisson(E_i e^{\theta_i})$$

$$\theta_i \sim \beta_0 + \mathrm{S}_i$$

$$\beta_0 \sim N(0{,}100000)$$

$$S_i \middle| \boldsymbol{S}_{\setminus i} \sim \mathcal{N}\left(\frac{\rho \sum_j w_{ij} S_j}{\rho \sum_j w_{ij} + 1 - \rho}, \frac{\sigma_S^2}{\rho \sum_j w_{ij} + 1 - \rho}\right) \text{ for } i = 1, \ldots, \mathcal{N}\text{areas}$$

$$\sigma_S^2 \sim \mathrm{InverseGamma}(1, 0.01),$$

$$\rho \sim \mathrm{Uniform}(0, 1)$$

Equation A 8

Neighbors:

In 2016 there were 2,310 SA2s defined for Australia, of which 18 had no spatial location. [3] A further 41 SA2s that had fewer than five residents on average per year during 2015-2019 were excluded. In addition, four very remote islands (Christmas Island, Cocos Island, Lord Howe Island and Norfolk Island) were not retained in the analyses. Estimates were thus modelled for 2,247 SA2s across Australia.

For a specific SA2, neighbors (adjacent areas) were all SA2s with a shared border. The default definition was refined so that the 13 island SA2s had at least one neighbor, generally the closest mainland SA2. Adjacency was symmetric, that is if area Y was a neighbor of area X then area X was also a neighbor of area Y. The binary, first-order symmetric adjacency weights matrix (with elements w_{ij}) required as an input into the spatial model was generated using the program GeoDa (version 1.14) [16] with 1st order queen adjacencies. [17, 18]

Computation

Convergence was assessed visually by trace and density plots of global parameters and selected spatial effects [18] and with the Geweke diagnostic test on all spatial effects. [19] The spatial effects were considered to have converged since less than 10% of areas had a significant Geweke diagnostic at the 5% level. Final models were fitted with a burn-in period of 100,000 iterations followed by 150,000 iterations with every tenth iteration kept for reporting results. Moran's I statistics were close to zero, ranging from -0.092 to -0.026 with non-significant p-values.

Sensitivity analyses

We used default priors in CARBayes package [20] for the spatial models. A sensitivity analysis was performed to ensure that estimates were not unduly influenced by prior distributions, especially the (inverse-Gamma) priors on the variance of the spatial random effect, σ_s^2. Comparison of convergence trace and density plots and spatial patterns indicated that the estimates were robust (results not shown) to prior choice. Moreover, multiple MCMC chains given different initial values were well mixed and gave similar results for the posterior estimates. The Gelman-Rubin statistics for modelled parameters were also very close to 1.0, indicative of good sampling. (13)

Posterior Probability

The posterior probability (PP) [21, 22] of the median smoothed SPR was defined [23] using Equation A 9 where $\mu_i^{(m)}$ is the mth MCMC sample for the log SPR estimate of area *i*:

$$PP_i = \frac{1}{M} \sum_{m=1}^{M} \prod \left(\exp\left(\mu_i^{(m)}\right) > 1 \right)$$

Equation A 9

Results were presented as maps in which green represented low PP values (<0.2) and suggested that screening participation rates were truly below average, conversely purple represented high PP (>0.8) indicating participation rates were truly above average. Values between 0.2 and 0.8 (pale grey) suggested a lack of evidence of a difference from the national average. [22]

Estimating missed screens

Consistent with previous survival studies, [24, 25] we set the optimum benchmark for bowel screening to be equal to the top 20th centile of the ranked smoothed SPRs. This corresponded to a smoothed SPR of 1.10, or a screening rate of 48%. The expected count in each of the lower 80% of ranked SA2s was multiplied by 1.10 to calculate the number of required screens to reach the threshold value. The number of missed screens due to spatial variation was then estimated as the difference between the number of required screens and the observed counts, summed over all these SA2s below the top 20th centile. Values stratified by broad geographical categories were also calculated.

References:

1. Australian Institute of Health and Welfare. Cancer screening programs: quarterly data Canberra: AIHW; 2022 [26 October 2022]. Available from: https://www.aihw.gov.au/reports/cancer-screening/national-cancer-screening-programs-participation/data.
2. Australian Institute of Health and Welfare. National Bowel Cancer Screening Program, Monitoring report 2022 Cat. No. CAN 148 Canberra: AIHW; 2022 [26 October 2022]. Pdf on internet]. Available from: https://www.aihw.gov.au/getmedia/3a53f195-5f73-4231-a0a7-c734fd2a0e98/aihw-can-148.pdf.aspx?inline=true.
3. Australian Bureau of Statistics. 1270.0.55.001 - Australian Statistical Geography Standard (ASGS): Volume 1 - Main Structure and Greater Capital City Statistical Areas, July 2016 Canberra: ABS; 2016 [09 November 2022]. Available from: http://www.abs.gov.au/ausstats/abs@.nsf/PrimaryMainFeatures/1270.0.55.001?OpenDocument.
4. Australian Cancer Atlas Version 09-2018. Cancer Council Queensland, Queensland University of Technology, Cooperative Research Centre for Spatial Information 2018 [09 November 2022]. Available from: https://atlas.cancer.org.au/.
5. Dasgupta P, Aitken JF, Condon J, Garvey G, Whop LJ, DeBats C, et al. Spatial and temporal variations in cervical cancer screening participation among indigenous and non-indigenous women, Queensland, Australia, 2008–2017. Cancer Epidemiol. 2020;69:101849. doi: 10.1016/j.canep.2020.101849.
6. Australian Bureau of Statistics. Australian Statistical Geography Standard (ASGS): Volume 1 - Main structure and greater capital city statistical areas, July 2011 Canberra: Australian Bureau of Statistics,; 2011 [09 November 2022]. Available from: https://www.abs.gov.au/AUSSTATS/abs@.nsf/allprimarymainfeatures/9593E06A9325683BCA257FED001561EA.
7. Australian Bureau of Statistics. Australian Statistical Geography Standard (ASGS) Correspondences 2016 [09 November 2022]. Available from: http://www.abs.gov.au/websitedbs/D3310114.nsf/home/Correspondences.
8. Australian Government Department of Health and Aged Care. National Bowel Cancer Screening Program-Policy Framework Canberra: Australian Government; 2017 [28 October 2022]. Pdf on internet]. Available from: https://www.health.gov.au/resources/publications/national-bowel-cancer-screening-program-policy-framework.
9. Australian Government Department of Health and Aged Care. National Bowel Cancer Screening Program-Participant's screening pathway Canberra: Australian Government; 2019 [28 October 2022]. Pdf on internet]. Available from: https://www.health.gov.au/sites/default/files/documents/2020/02/national-bowel-cancer-screening-program-participant-screening-pathway-national-bowel-cancer-screening-program-participant-s-screening-pathway.pdf.
10. Australian Bureau of Statistics. Regional Population by Age and Sex, 2020 Canberra: ABS; 2021 [09 November 2022]. Available from: https://www.abs.gov.au/statistics/people/population/regional-population-age-and-sex/2020#data-download.
11. Australian Bureau of Statistics. National, state and territory population, December 2021 Canberra: ABS; 2022 [09 November 2022]. Available from: https://www.abs.gov.au/statistics/people/population/national-state-and-territory-population/latest-release#notes.
12. Dobson AJ. An introduction to generalized linear models. Fourth edition. ed. Barnett AG, editor. Boca Raton, FL: CRC Press, Taylor & Francis Group; 2018.
13. Australian Bureau of Statistics. Census of Population and Housing: Socio-Economic Indexes for Areas (SEIFA), cat. No. 2033.0.55.001. Canberra: ABS; 2018 [09 November 2022]. Available

from:
https://www.abs.gov.au/AUSSTATS/abs@.nsf/DetailsPage/2033.0.55.0012016?OpenDocument.
14. Australian Bureau of Statistics. Australian Statistical Geography Standard (ASGS): Volume 5 - Remoteness Structure, July 2016, cat. No.1270..0.55.005: ABS; 2018 [09 November 2022]. Available from:
https://www.abs.gov.au/AUSSTATS/abs@.nsf/Latestproducts/1270.0.55.005Main%20Features15July%202016.
15. Leroux BG, Lei X, Breslow N. Estimation of disease rates in small areas: a new mixed model for spatial dependence. In: Halloran ME, Berry D, editors. Statistical models in epidemiology, the environment and clinical trials. New York: Springer; 2000. p. 135-78.
16. Anselin L. GeoDa 1.14 2018 [10 February 2020]. Available from:
https://geodacenter.github.io/.
17. Anselin L. Contiguity-Based Spatial Weights University of Chicago, Center for Spatial Data Science2018 [10 February 2020]. Available from:
https://geodacenter.github.io/workbook/4a_contig_weights/lab4a.html.
18. Duncan EW, Cramb SM, Aitken JF, Mengersen KL, Baade PD. Development of the Australian Cancer Atlas: spatial modelling, visualisation, and reporting of estimates. Int J Health Geogr. 2019;18(1):21. doi: 10.1186/s12942-019-0185-9. PubMed PMID: 31570101; PubMed Central PMCID: PMCPMC6771109.
19. Geweke J. Evaluating the accuracy of sampling-based approaches to the calculation of posterior moments. In: Bernardo JM, Berger J, Dawid AP, Smith AFM, editors. Bayesian Statistics 4. Oxford: Oxford University Press; 1992. p. 169-93.
20. Lee D. CARBayes version 5.2.5: An R Package for Spatial Areal Unit Modelling with Conditional Autoregressive Priors Glasgow: University of Glasgow; 2021 [09 November 2022]. Available from: https://cran.r-project.org/web/packages/CARBayes/CARBayes.pdf.
21. Cramb SM, Mengersen KL, Baade PD. Spatio-temporal relative survival of breast and colorectal cancer in Queensland, Australia 2001-2011. Spat Spatiotemporal Epidemiol. 2016;19:103-14. doi: 10.1016/j.sste.2016.08.002.
22. Richardson S, Thomson A, Best N, Elliott P. Interpreting posterior relative risk estimates in disease-mapping studies. Environ Health Perspect. 2004;112(9):1016-25. doi: 10.1289/ehp.6740. PubMed PMID: 15198922; PubMed Central PMCID: PMCPMC1247195.
23. Cramb SM, Duncan EW, Aitken JF, Soyer HP, Mengersen KL, Baade PD. Geographical patterns in melanoma incidence across Australia: can thickness differentials reveal the key drivers? Ann Cancer Epidemiol. 2020;4:11. doi: 10.21037/ace-20-13.
24. Cramb SM, Mengersen KL, Turrell G, Baade PD. Spatial inequalities in colorectal and breast cancer survival: premature deaths and associated factors. Health & Place. 2012;18(6):1412-21. doi: 10.1016/j.healthplace.2012.07.006. PubMed PMID: 22906754.
25. Yu XQ, O'Connell DL, Gibberd RW, Smith DP, Dickman PW, Armstrong BK. Estimating regional variation in cancer survival: a tool for improving cancer care. Cancer Causes Control. 2004;15(6):611-8. doi: 10.1023/B:CACO.0000036165.13089.e8. PubMed PMID: 15280640.
