# Supplementary material for: Geographical and spatial variations in bowel cancer screening participation, Australia, 2015–2020
Source: PLoS One. 2023 Jul 20;18(7):e0288992. doi: 10.1371/journal.pone.0288992 (PMC10358922; doi:10.1371/journal.pone.0288992)
Supplement: S2 Appendix — (PDF) [file pone.0288992.s002.pdf]

S2 Appendix: Example R codes

Model for screening participation

```
# Required packages
	library(CARBayes) # for S.CARLeroux()
	library(spdep) # For nb2mat()
	library(tidyverse) # For as_tibble
	library(reshape) # For melt
# Reading in neighbourhood matrix data file for Australia
	aust<-read.gal("SA2_2016Aust_grid.gal")
	class(aust)
# where aust is a neighbourhood matrix (i.e. class = nb)
	W <- nb2mat(aust, style = "B")
# Bowel is the input data set used for spatial modelling
	bowel<-read.csv("bowel)
	data<-as_tibble(bowel)
# Input data set contains observed count (count), expected (expect) and id (the
region number from 1 to x where x is the total number of regions. The region id
numbers must match the ones in the matrix W.
	data.input = data.frame(obs = data$count,
	id = data$grid)
# Setting up the spatial model
	MCMC <- S.CARleroux(
	formula <-obs~offset(log(expect)),
	data =data.input,
	family = "poisson",
	prior.tau2 = c(1, 0.01), # Shape and scale
	rho = NULL,
	W = W,
	burnin = 50000,
	n.sample = 150000,
	thin = 10)
# Generate matrix of fitted values for number of screened people
	fitted <- MCMC$samples$fitted
# Divide fitted values by expected counts to calculate SPR
	SPR <- ( t( t( fitted ) /data.input$expect ) )
	SPR_ <- as.data.frame(SPR)
	SPR_ <- cbind(1:nrow(SPR_),SPR_)
	names(SPR_)[1]<-"id"
	SPRlong <- melt(SPR_, id = c("id"))
	SPRlong$grid <- as.numeric(substring(SPRlong$variable,2))
	names(SPRlong)[3] <- "SPR"
	SPRlong$pptothi <- as.numeric(SPRlong$SPR > 1)
```

```
# Create data set with median SPR and credible intervals for export
      SPRgroup <- group_by(SPRlong,grid)
      SPRsumm <- summarise(
      sirgroup,
      SPR_p50 = median(SPR),
      SPR_p10 = quantile(SPR, 0.10),
      SPR_p90 = quantile(SPR, 0.90),
      SPR_p025 = quantile(SPR,0.025),
      SPR_p975 = quantile(SPR,0.975),
      pptothi = sum(pptothi),
      )
# Calculate PP-the posterior probability that smoothed SPR is greater than one
      SPRsumm$pphi <- SPRsumm$pptothi/nrow(fitted)
# Export data to a .csv file
      write.csv(SPRsumm, file = "modelSPR.csv", row.names = FALSE)
# Reconstructing count
      counts <- fitted
      count = apply(counts, 2, median))
# Clip count to preserve confidentiality for numbers <3
      count.clip = count
      count1.clip[count <= 3] = 3
# export count data
      exportc <- data.frame(count = count.clip,
      id = data$grid,
      sa2 = data$sa2
      )
# Export count data to a .csv file
      write.csv(SPRsumm, file = "count.csv", row.names = FALSE)
```

Mapping code- used to create maps using shapefiles in conjunction with ggplot2

```
# Required packages
      library(sf) # for working with shape files
      library(ggplot2) # for making maps with ggplot2
      library(gridExtra) # For grid.arrange()
      library(scales) # For rescale()
      library(dplyr) # For inner_join()
# Read in the shapefile for Australia based on 2016 SA2 boundaries
      map <- st_read ("SA2_2016_AUST.shp")
# Fortify map
      map.df <- fortify(map)
# Read in shape file for Australian states and territories
      mapbor <- st_read ("STE_AUST.shp")
# Read in data to be mapped such as smoothed standardized participation ratio
      bowel<- read.csv ("modelSPR.csv")
# Define modelled parameter to be mapped such as SPR
      SPR.smth <- bowel$SPR_p50
      SPR.smth[which(is.nan(SPR.smth))] <- 0
      SPR.clip <- SPR.smth
# For clarity, truncate extreme SPR values
      cut.offs <- c(1/1.5, 1.5)
      SPR.clip[which(SPR.clip < cut.offs[1], arr.ind = TRUE)] <- cut.offs[1]
      SPR.clip[which(SPR.clip > cut.offs[2], arr.ind = TRUE)] <- cut.offs[2]
# Define fill colours as hexadecimal values (change as desired)
      Fill.colours <- c("#2C7BB6", "#2C7BB6", "#ABD9E9", "#FFFFBF", "#FDAE61",
      "#D7191C", "#D7191C")
      Breaks.fill <- c(1/1.5, 1/1.25, 1, 1.25, 1.5)
# Values corresponding to the fill colours
      log.cut.offs <- log2(cut.offs)
      End <- log2(1.6)
      Fill.values <- c(-End, log.cut.offs[1], log.cut.offs[1] * 0.5, 0,
      log.cut.offs[2] * 0.5, log.cut.offs[2], End)
# Note: we use logged values for the SPR so that the colour gradient is linear
# Rescaled fill values
      Fill.values.r <- rescale(Fill.values, from = range(Fill.values), na.rm =
      TRUE)
# Create data frame of parameters for ggplot- will need to be appended to the
shapefile
      Append <- data.frame(
      SA2_5DIG16 =  factor(bowel$sa2),
      SPR = SPR.clip
      )
# Note we use factor to allow the two data frames to be merged using inner join
# Merge values with shapefile data frame
      dat<- left_join(map.df, Append, by = "SA2_5DIG16")
```

```
# Set up basic components of the plot
      gg.base <- ggplot(data = NULL) +
      ggtitle("") +
      coord_sf() +
      theme_void() +
      theme(
      legend.position = "bottom",
      plot.title = element_text(size =12, margin = margin(0,0,2,0)),
      plot.margin = unit(c(1,1,1,1), "mm")
      ) +
      guides(fill = guide_colourbar(barwidth = 10 ))
# Add visible layers for fill colors
      gg.SPR <- gg.base +
      geom_sf(data = dat, size = 0.1, color="NA",aes(fill =log2(SPR))) +
      geom_sf(data = mapbor, colour = "black", fill = NA) +
      scale_fill_gradientn("", colours = Fill.colours, values = Fill.values.r ,
      labels = as.character(round(Breaks.fill, 3)),
      breaks = log2(Breaks.fill), limits = range(Fill.values)) +
      scale_y_continuous(expand = c(0.01, 0.01))
# Render plots
      ggsave("Australia SPR.png", width = 7, height =4, dpi=300, bg ="WHITE")

# Define city inset limits (determined by trial and error)
      lims <- data.frame(
      xmin = c(152.6, 150.35, 144.5, 115.45, 138.2, 146.9, 148.8, 130.4),
      xmax = c(153.6, 151.35, 145.5, 116.45, 138.8, 147.5, 149.3, 131.0),
      ymin = -c(28, 34.4, 38.4, 32.5, 35, 43.2, 35.4, 12.7),
      ymax = -c(27, 33.4, 37.4, 31.5, 34.4, 42.7, 34.8, 12.2),
      city = c("Brisbane", "Sydney", "Melbourne", "Perth", "Adelaide", "Hobart",
      "Canberra", "Darwin")

# Create Australia map with annotated insets for major cities
      gg.SPRi <- gg.SPR +
      annotate("rect",
      xmin = lims$xmin - 0.1, xmax = lims$xmax + 0.1,
      ymin = lims$ymin - 0.1, ymax = lims$ymax + 0.1,
      colour = "black", fill = NA)
# Create base plot for Australia for inset maps
      gg.i <- gg.SPR +
      theme_void() +
      theme(
      legend.position = "none",
      plot.title = element_text(size = 6, margin = margin(0,0,2,0)) ,
      plot.margin = unit(c(1,1,1,1), "mm")
      ) +
      guides(fill = guide_colourbar(barwidth = 10)
      )
```

```
      gg.base.inset <- gg.i + guides(scale = "NONE")
# Create inset maps
      gg.inset <- vector("list", 8)

for (i in 1:nrow(lims)) {
      gg.inset[[i]] <- gg.base.inset +
      xlim(lims$xmin[i], lims$xmax[i]) +
      ylim(lims$ymin[i], lims$ymax[i]) +
      ggtitle(label = lims$city[i])
      }
# Render plots
      layout <- rbind(c(8,9,9,1),
      c(4,9,9,2),
      c(5,3,6,7)
      )

      comb <- grid.arrange(grobs = list(gg.inset[[1]], gg.inset[[2]],
      gg.inset[[3]], gg.inset[[4]], gg.inset[[5]], gg.inset[[6]], gg.inset[[7]],
      gg.inset[[8]], gg.SPRi),
      layout_matrix = layout)
# Export plots
      ggsave("Australia_SPR_inset.png", plot = comb, width = 7, height =4, dpi
      =300)
```
