# Supplementary material for: Geographical and spatial variations in bowel cancer screening participation, Australia, 2015–2020
Source: PLoS One. 2023 Jul 20;18(7):e0288992. doi: 10.1371/journal.pone.0288992 (PMC10358922; doi:10.1371/journal.pone.0288992)
Supplement: S1 Table — (PDF) [file pone.0288992.s004.pdf]

S1 Table Participation rate ratios (PRR) for bowel cancer screening, from multivariable generalized Poisson models persons, Australia, 2019-2020

Variable	Participation rate ratio (PRR) [95% confidence intervals] [a,b,c]
State/territory	*p*<0.001
New South Wales	1.00
Victoria	1.11 [0.97, 1.24]
Queensland	1.02 [0.91, 1.15]
South Australia	1.24 [1.04, 1.48]
Western Australia	1.12 [0.96, 1.32]
Tasmania	1.17 [0.93, 1.47]
Northern Territory	0.64 [0.47, 0.86]
Australian Capital Territory	1.06 [0.85, 1.31]
Remoteness[d]	*p*=0.001
Major cities	1.00
Inner regional	1.12 [1.01, 1.26]
Outer regional	1.09 [0.94, 1.25]
Remote	0.75 [0.59, 0.95]
Area-level disadvantage[e]	*p*=0.02
Most advantaged	1.00
Q4	0.94 [0.82, 1.07]
Q3	0.91 [0.79, 1.05]
Q2	0.88 [0.76, 1.02]
Most disadvantaged	0.79 [0.69, 0.92]

a. The 2-year period covered the calendar year from 1 January 2019 to 31 December 2020 in the following year.

b. Estimated using multivariable generalised linear negative binomial models adjusted for all variables in the Table with the outcome being the number of screened persons and the offset being the expected counts.

c. Wald's joint test of coefficients for multivariable generalised linear negative binomial models.

d. Remote areas were defined by the Remoteness Areas 2016 classification with remote and very remote areas combined.

e. Area-level disadvantage was defined by the 2016 SEIFA Index of Relative Socioeconomic Advantage and Disadvantage
