# Supplementary material for: Geographical and spatial variations in bowel cancer screening participation, Australia, 2015–2020
Source: PLoS One. 2023 Jul 20;18(7):e0288992. doi: 10.1371/journal.pone.0288992 (PMC10358922; doi:10.1371/journal.pone.0288992)
Supplement: S2 Table — (PDF) [file pone.0288992.s005.pdf]

S2 Table Number of missed bowel cancer screens, Australia, 2019-2020

Variable	Number of screens[a,b]	Number of missed screens[a,c]	Row %
State/territory			
New South Wales	742,775	113,365	15.3
Victoria	641,003	29,040	4.5
Queensland	469,104	79,517	17.0
South Australia	206,071	6,604	3.2
Western Australia	264,641	14,166	5.4
Tasmania	67,791	2,767	4.1
Northern Territory	10,295	7,334	71.2
Australian Capital Territory	41,215	1,408	3.4
Remoteness[4]			
Major cities	1,651,214	174,553	10.6
Inner regional	540,859	37,345	6.9
Outer regional	223,183	31,553	14.1
Remote	27,639	10,750	38.9
Area-level disadvantage[e]			
Most advantaged	540,109	26,397	4.9
Q4	484,437	37,487	7.7
Q3	509,605	52,467	10.3
Q2	493,834	55,693	11.3
Most disadvantaged	413,835	82,076	19.8
Total	2,442,895	254,201	10.4

a. The 2-year period covered the calendar year from 1 January 2019 to 31 December 2020 in the following year.

b. Modelled counts of screens.

c. Estimated using threshold value of Standard Participation ratio (SPR) of 1.10, the minimum value of the median smoothed estimate for top 20% of SA2s and calculating the number of additional screens required if remaining 80% of SA2s were to have an SPR of 1.10 as described in text.

d. Remote areas were defined by the Remoteness Areas 2016 classification with remote and very remote areas combined.

e. Area-level disadvantage was defined by the 2016 SEIFA Index of Relative Socioeconomic Advantage and Disadvantage
