# Supplementary material for: Geographical and spatial variations in bowel cancer screening participation, Australia, 2015–2020
Source: PLoS One. 2023 Jul 20;18(7):e0288992. doi: 10.1371/journal.pone.0288992 (PMC10358922; doi:10.1371/journal.pone.0288992)
Supplement: S3 Table — (PDF) [file pone.0288992.s006.pdf]

S3 Table Estimated resident population (ERP) for each capital city, Australia, 2021

City	ERP as of 30 June 2021[a,b]	% of total population
Sydney	5,259,764	20.4
Melbourne	4,976,157	19.3
Brisbane	2,568,927	10.0
Adelaide	1,402,393	5.4
Perth	2,192,229	8.5
Hobart	251,047	1.0
Darwin	148,801	0.6
Canberra	453,558	1.8
Total capital cities	17,252,876	67.0
Total Australia	25,739,256	

a. Estimated resident population for persons of all ages as of 30 June 2021.

b. Source: Australian Bureau of Statistics, Regional population 20221. Available from: https://www.abs.gov.au/statistics/people/population/regional-population/latest-release.
