# Supplementary figures and images for: Geographical and spatial variations in bowel cancer screening participation, Australia, 2015–2020
Source: PLoS One. 2023 Jul 20;18(7):e0288992. doi: 10.1371/journal.pone.0288992 (PMC10358922; doi:10.1371/journal.pone.0288992)

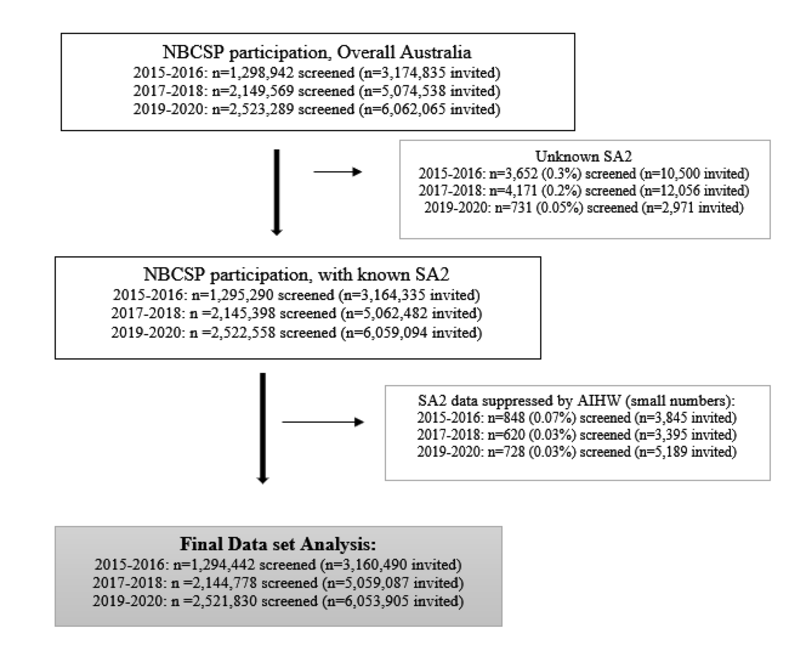

Supplement: S1 Fig — Abbreviations are SA2 (statistical area level 2), NBCSP (National Bowel Cancer Screening Program), AIHW (Australian Institute of Health and Welfare). (TIF) [file pone.0288992.s007.tif]

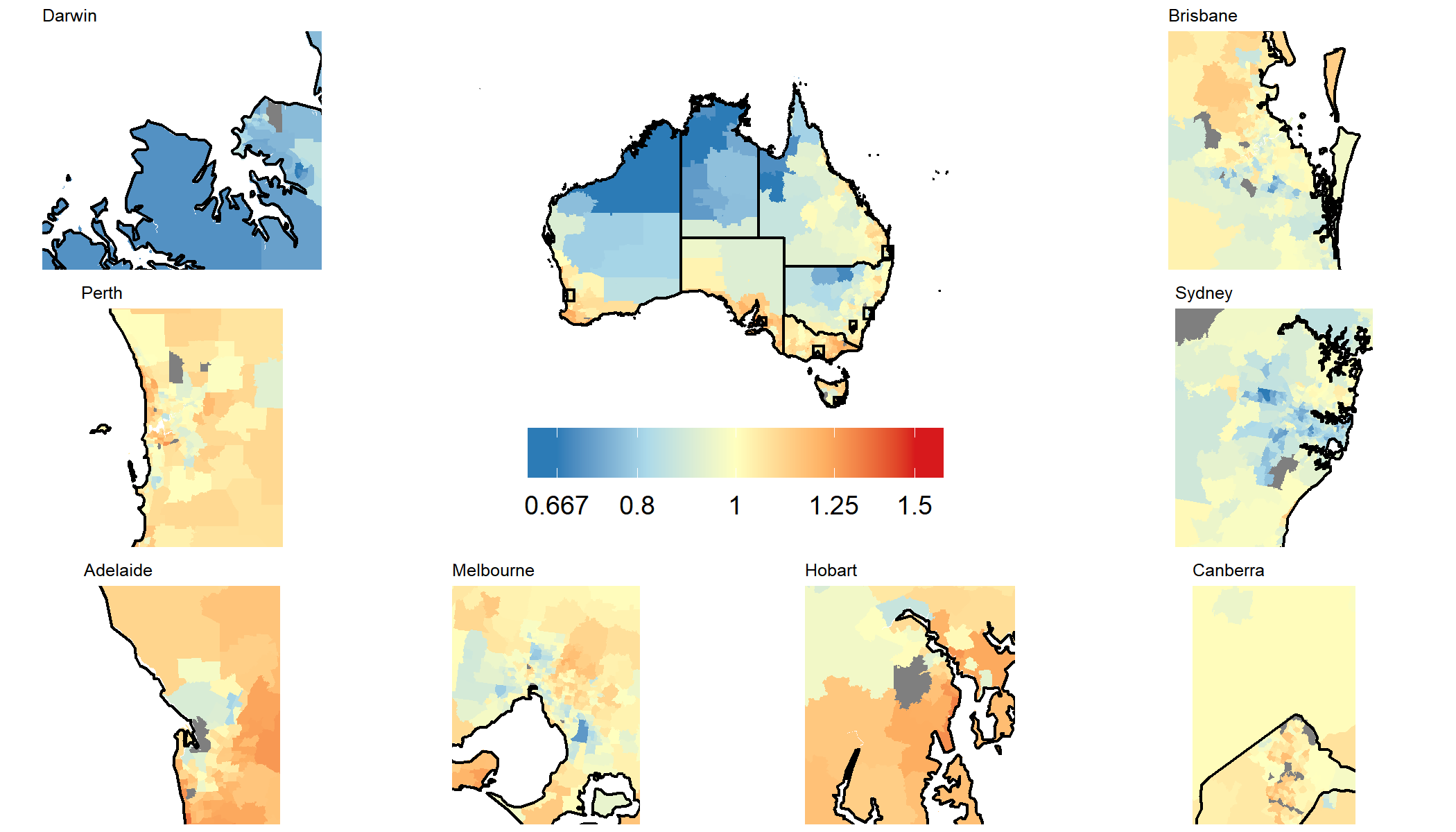

Supplement: S2 Fig — The map for Canberra includes the boundary between the Australian Capital Territory and New South Wales. An SPR with value 1 indicates that screening participation is the same as the national average (40.9%) during 2015–2016. (TIF) [file pone.0288992.s008.tif]

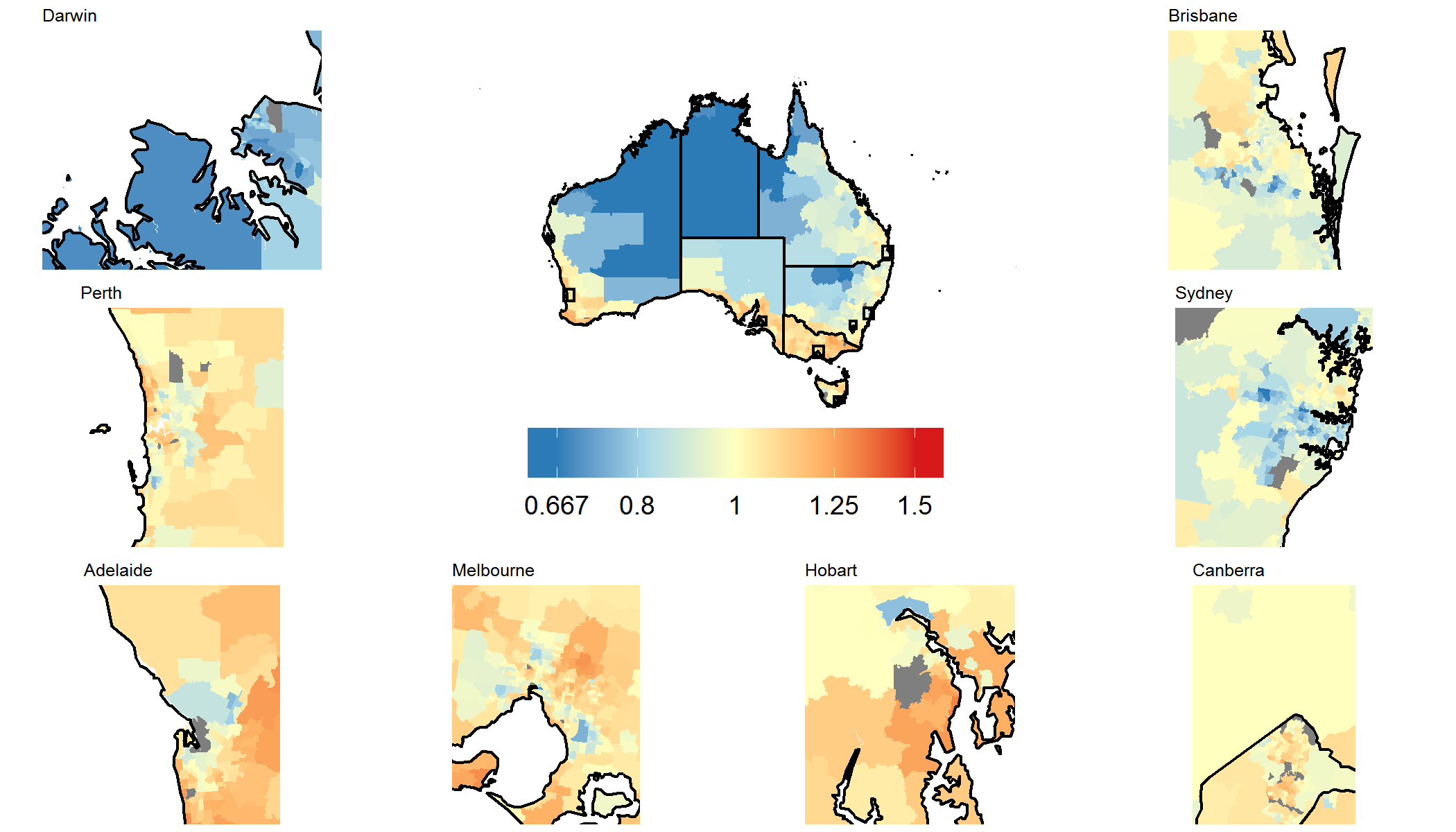

Supplement: S3 Fig — The map for Canberra includes the boundary between the Australian Capital Territory and New South Wales. An SPR with value 1 indicates that screening participation is the same as the national average (40.9%) during 2015–2016. (TIF) [file pone.0288992.s009.tif]

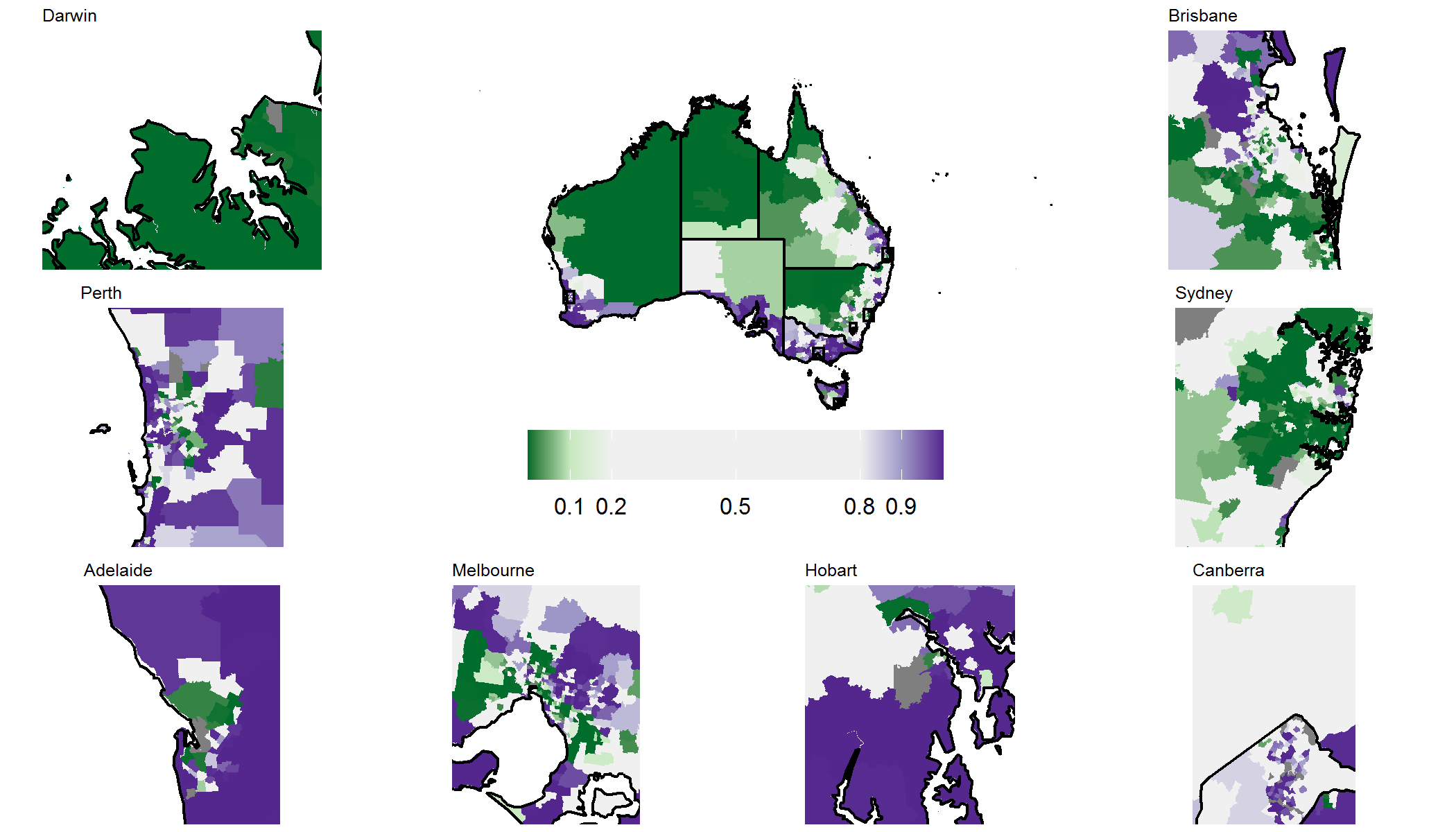

Supplement: S4 Fig — Values of the PP for smoothed standardised participation ratios are truly lower than average (PP <0.2), uncertain (PP = 0.2–0.8) and higher than average (PP >0.8). The map for Canberra includes the boundary between the Australian Capital Territory and New South Wales. (TIF) [file pone.0288992.s010.tif]

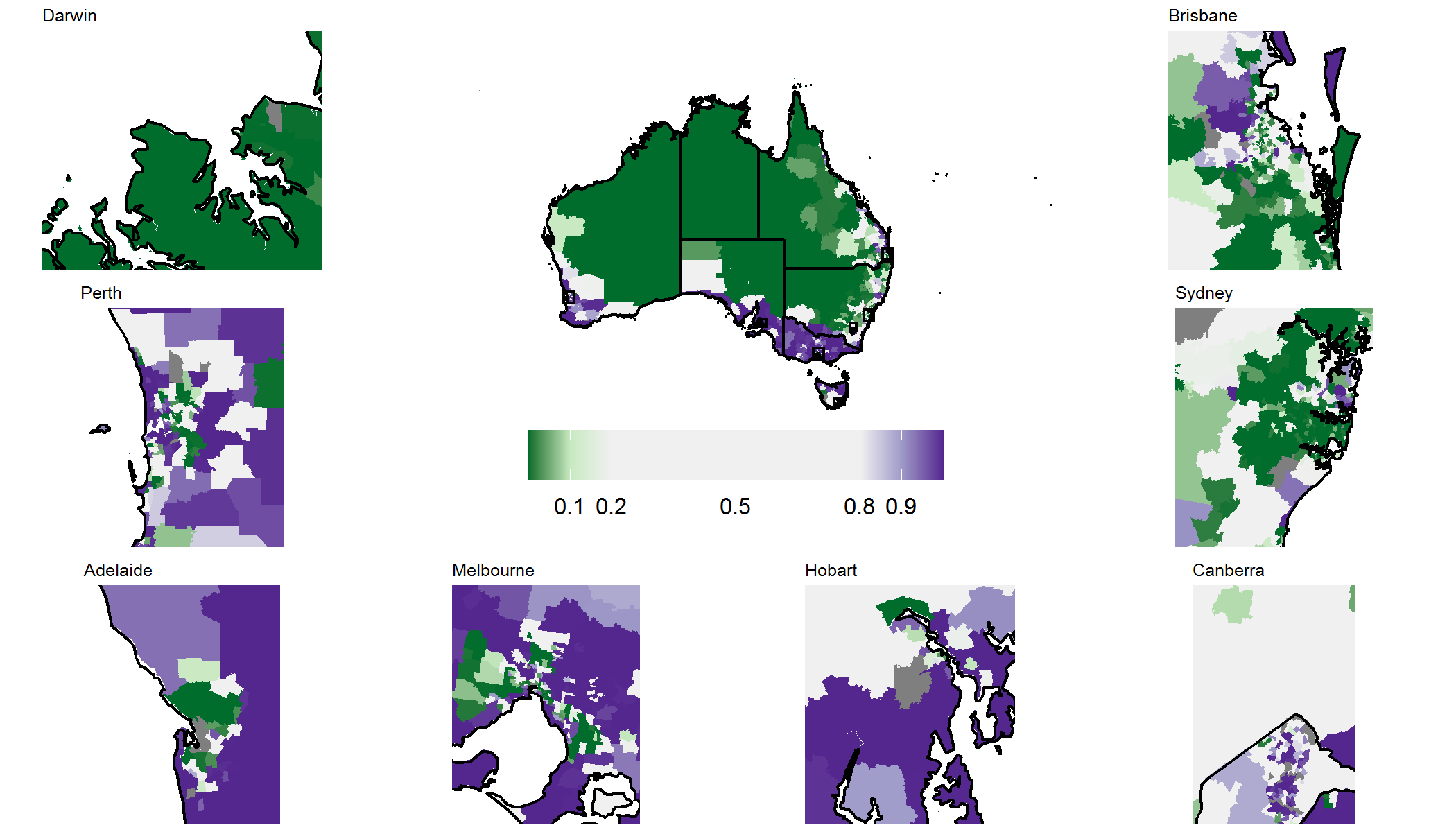

Supplement: S5 Fig — Values of the PP for smoothed standardised participation ratios are truly lower than average (PP <0.2), uncertain (PP = 0.2–0.8) and higher than average (PP >0.8). The map for Canberra includes the boundary between the Australian Capital Territory and New South Wales. (TIF) [file pone.0288992.s011.tif]

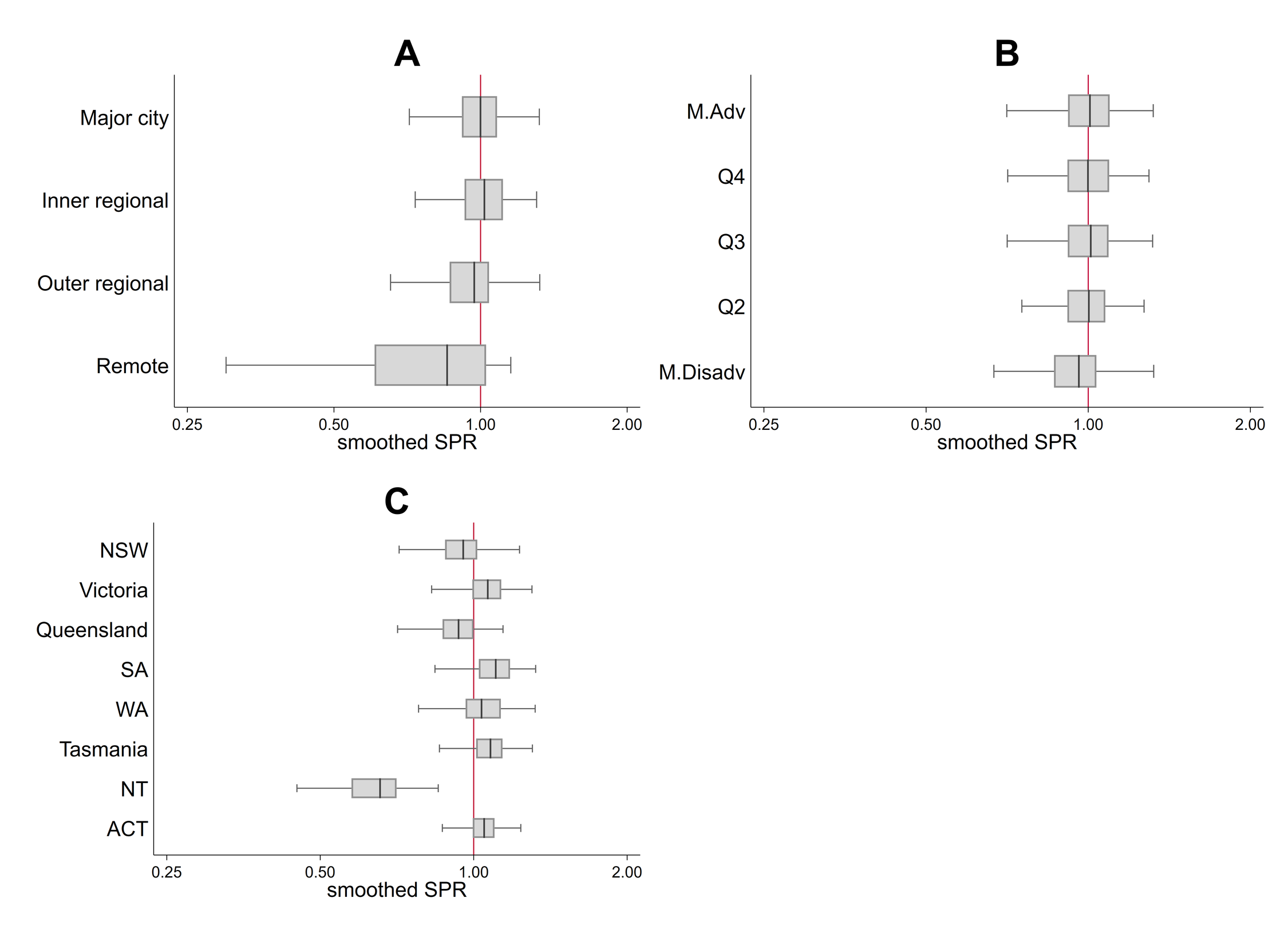

Supplement: S6 Fig — The vertical line indicates an SPR of 1 representing the national average. Abbreviations are M.Adv Most advantaged, M.Disadv Most disadvantaged, NSW: New South Wales, WA: Western Australia, SA: South Australia, ACT: Australian Capital Territory, NT Northern Territory. (TIF) [file pone.0288992.s012.tif]

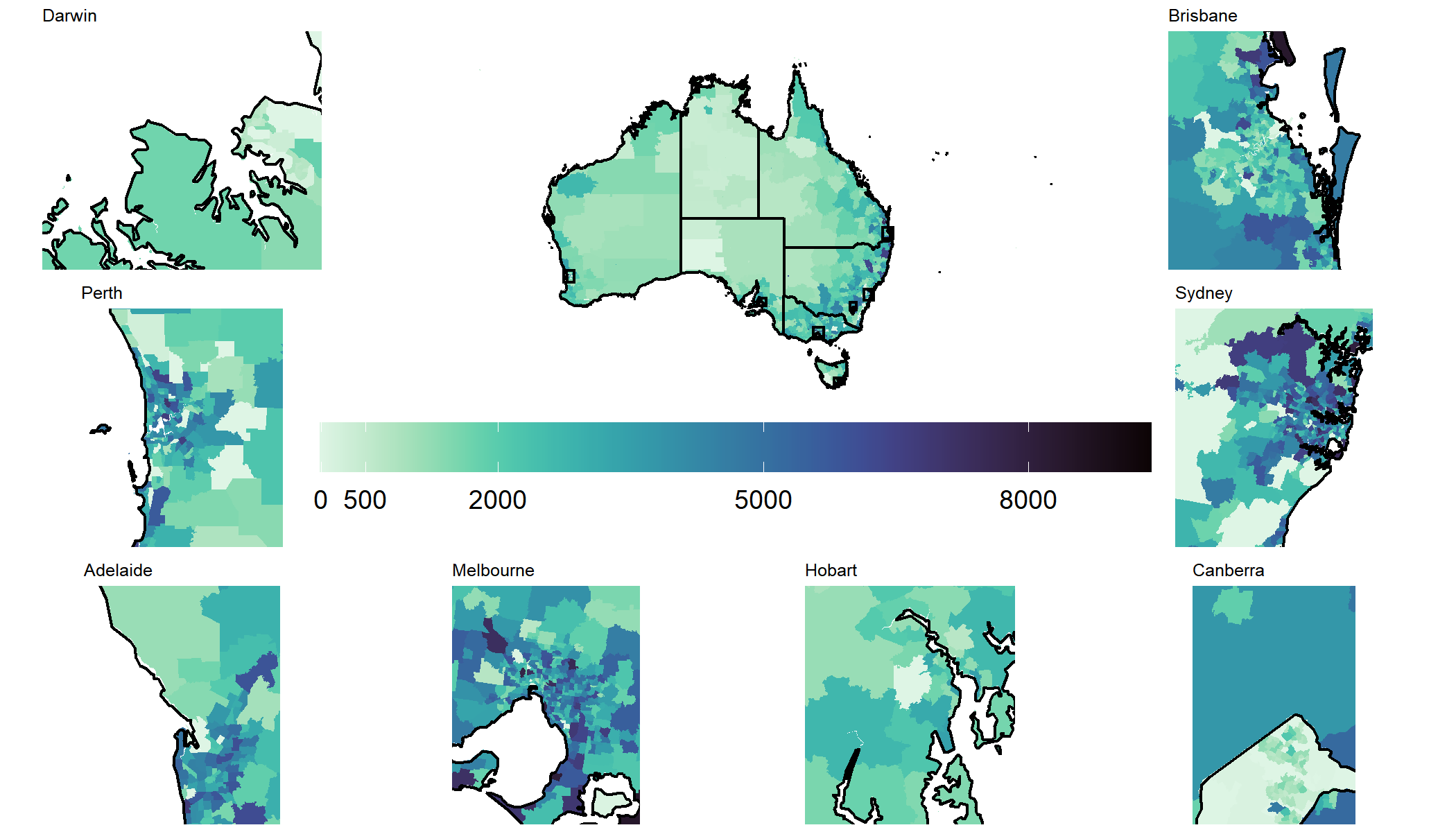

Supplement: S7 Fig — The map for Canberra includes the boundary between the Australian Capital Territory and New South Wales. (TIF) [file pone.0288992.s013.tif]

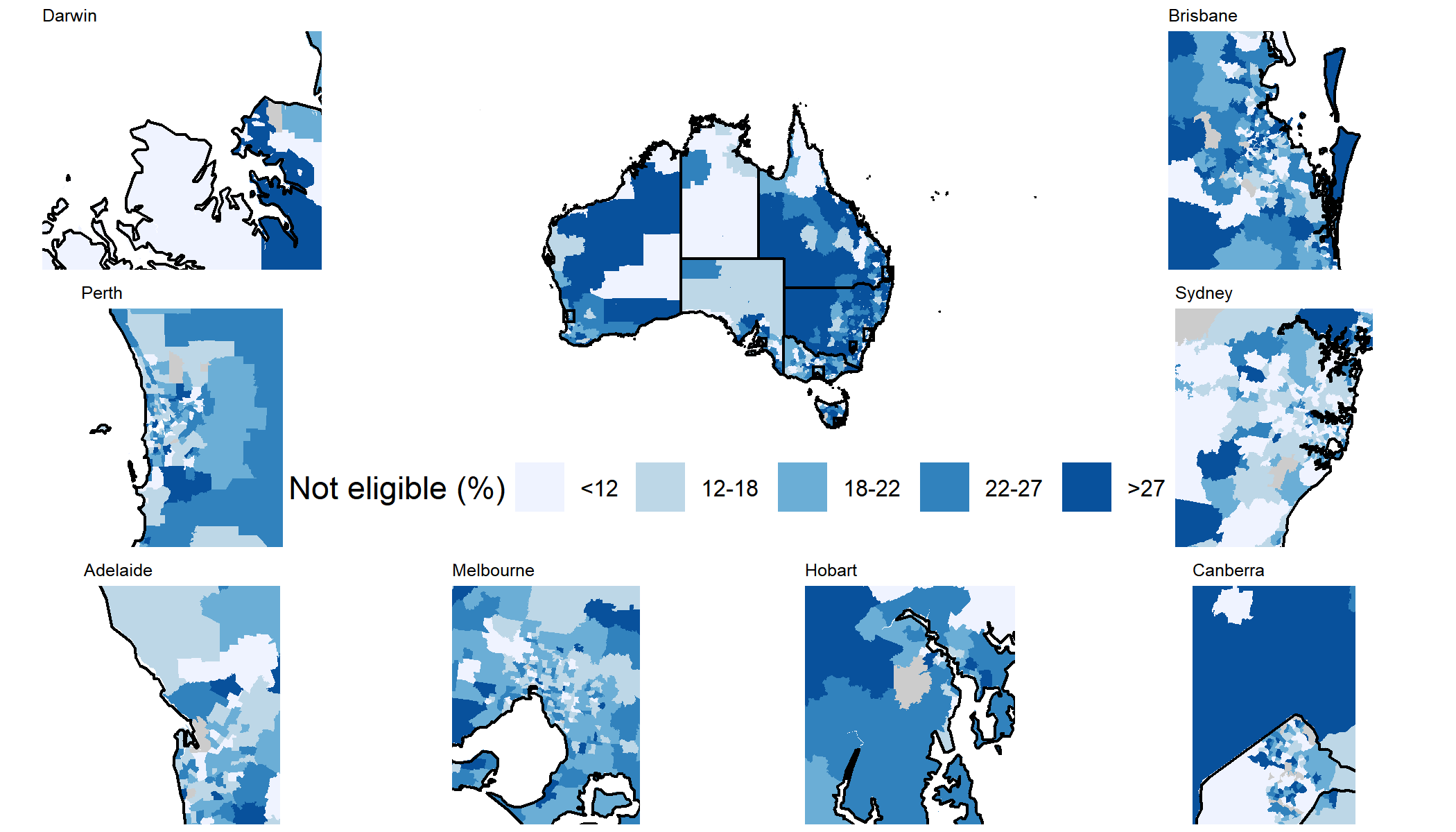

Supplement: S8 Fig — The map for Canberra includes the boundary between the Australian Capital Territory and New South Wales. (TIF) [file pone.0288992.s014.tif]
